# Global immune characterization of HBV/HCV-related hepatocellular carcinoma identifies macrophage and T-cell subsets associated with disease progression

**DOI:** 10.1038/s41421-020-00214-5

**Published:** 2020-12-08

**Authors:** Guohe Song, Yang Shi, Meiying Zhang, Shyamal Goswami, Saifullah Afridi, Lu Meng, Jiaqiang Ma, Yi Chen, Youpei Lin, Juan Zhang, Yuming Liu, Zijie Jin, Shuaixi Yang, Dongning Rao, Shu Zhang, Aiwu Ke, Xiaoying Wang, Ya Cao, Jian Zhou, Jia Fan, Xiaoming Zhang, Ruibin Xi, Qiang Gao

**Affiliations:** 1 Department of Liver Surgery and Transplantation, Liver Cancer Institute, Zhongshan Hospital, and Key Laboratory of Carcinogenesis and Cancer Invasion (Ministry of Education), Fudan University, Shanghai 200032, China; 2 Peking-Tsinghua Center for Life Sciences, Academy for Advanced Interdisciplinary Studies, Peking University, Beijing 100871, China; 3 The Center for Microbes, Development and Health, Key Laboratory of Molecular Virology & Immunology, Institut Pasteur of Shanghai, Chinese Academy of Sciences, Shanghai 200031, China; 4 Department of Biological Sciences (DBS), National University of Medical Sciences (NUMS), Secretariat c/o Military Hospital, Adjacent to Armed Force Institute of Cardiology, The Mall Rawalpindi, Rawalpindi 46000, Pakistan; 5 Department of Laboratory Medicine, Nantong First People’s Hospital, The Second Affiliated Hospital of Nantong University, Nantong, Jiangsu 226001 China; 6 School of Mathematical Sciences, Peking University, 5 Yiheyuan Road, Beijing 100871, China; 7 Cancer Research Institute, Xiangya School of Medicine, Central South University, Changsha, Hunan 410078 China; 8 Key Laboratory of Medical Epigenetics and Metabolism, Institutes of Biomedical Sciences, Fudan University, Shanghai 200032, China; 9 School of Mathematical Sciences and Center for Statistical Science, Peking University, Beijing 100871, China; 10 State Key Laboratory of Genetic Engineering, Fudan University, Shanghai 200433, China

**Keywords:** Tumour immunology, Liver cancer, Tumour immunology, Tumour immunology, Liver cancer

## Abstract

Diverse immune cells in the tumor microenvironment form a complex ecosystem, but our knowledge of their heterogeneity and dynamics within hepatocellular carcinoma (HCC) still remains limited. To assess the plasticity and phenotypes of immune cells within HBV/HCV-related HCC microenvironment at single-cell level, we performed single-cell RNA sequencing on 41,698 immune cells from seven pairs of HBV/HCV-related HCC tumors and non-tumor liver tissues. We combined bio-informatic analyses, flow cytometry, and multiplex immunohistochemistry to assess the heterogeneity of different immune cell subsets in functional characteristics, transcriptional regulation, phenotypic switching, and interactions. We identified 29 immune cell subsets of myeloid cells, NK cells, and lymphocytes with unique transcriptomic profiles in HCC. A highly complex immunological network was shaped by diverse immune cell subsets that can transit among different states and mutually interact. Notably, we identified a subset of M2 macrophage with high expression of CCL18 and transcription factor CREM that was enriched in advanced HCC patients, and potentially participated in tumor progression. We also detected a new subset of activated CD8^+^ T cells highly expressing XCL1 that correlated with better patient survival rates. Meanwhile, distinct transcriptomic signatures, cytotoxic phenotypes, and evolution trajectory of effector CD8^+^ T cells from early-stage to advanced HCC were also identified. Our study provides insight into the immune microenvironment in HBV/HCV-related HCC and highlights novel macrophage and T-cell subsets that could be further exploited in future immunotherapy.

## Introduction

Hepatocellular carcinoma (HCC) is the fourth leading cause of cancer death worldwide, with chronic hepatitis B (HBV) and C (HCV) virus infection as the leading risk factors^1^. Recent immune therapies, including inhibitors blocking immune checkpoints, have shown encouraging clinical results in HCC. However, treatment outcomes vary among patients and achieve only about a 20% response rate^2,3^. HCC is known as an inflammation-driven disease, and it is rational that the quality and quantity of immune cell infiltrations and dynamic interactions may significantly impact on the efficacy of immunotherapy^4^.

Tumor microenvironment (TME) is a complex and heterogeneous ecosystem. Innate immune cells, such as the classically activated macrophages (M1) can kill and remove tumor cells, while the M2 macrophages, also considered as tumor-associated macrophages (TAMs), promote tumor progression^5^. Although it is currently clear that macrophages play a dual role in tumor immune responses, the heterogeneity, functional characteristics, and relationship between M1 and M2 macrophages still need further characterization. Also, various subsets of adaptive immune cells in TME display considerable plasticity in anti-tumor response. For example, CD8^+^ cytotoxic T cells play a critical role in tumor control and contribute to a better prognosis for HCC^6^. However, these cells could display an exhausted state by persistent antigen stimulation and display a compromised capacity to produce pro-inflammatory cytokines^7^. Thus, comprehensive characterization of diverse immune subsets will bring new clues for developing tumor immunotherapy.

Single-cell RNA-sequencing (scRNA-seq) has allowed for comprehensive profiling of the immune system in an unprecedented way^8^. Applying scRNA-seq on immune cell populations has identified novel immune subsets in many types of tumors, including lung^9,10^, colorectal^11^, liver^12,13^, and breast cancer^14^. Here, we conducted a comprehensive scRNA-seq of immune cells without filtering for cell type markers from seven HBV/HCV-related HCC patients. We discovered a considerable spatiotemporal heterogeneity and plasticity of immune subsets. Importantly, we detected and validated a new subset of CCL18^+^ M2 macrophages and a new subset of XCL1^+^CD8^+^ T cells that correlated with disease progression and anti-tumor responses, respectively. Our findings provided a valuable resource for deeper understanding of HBV/HCV-related HCC immunology, and may guide future immunotherapeutic strategies.

## Results

### Single-Cell Profiling of immune cells in HCC microenvironment

**Fig. 1:**
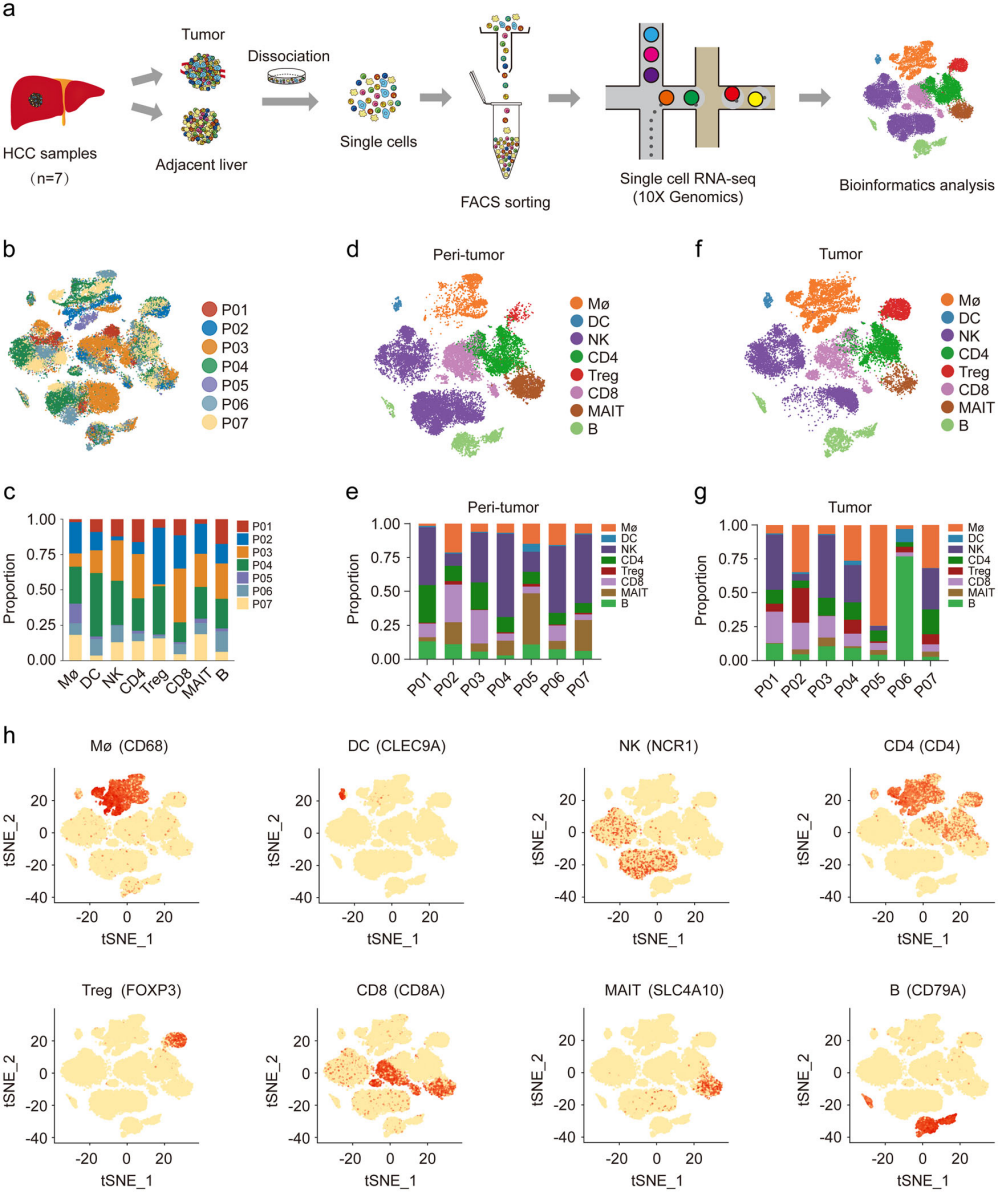
Single-Cell Profiling of diverse immune cells from HCC tumors and distal peri-tumors. **a** Overview of the study workflow. **b**, **c** t-SNE plot and proportions of all 41,698 cells annotated by the seven patients. **d**, **e** t-SNE plot and proportions of cell types vary across sample origin from peri-tumor tissues. **f**, **g** t-SNE plot and proportions of cell types vary across sample origin from HCC tumor. **h** Expression of cell-type-specific marker genes illustrated in t-SNE plots.

We performed scRNA-seq on immune cells isolated from seven treatment-naïve HCC (P01~P07) and adjacent non-tumor liver tissues (Fig. 1a–c; Supplementary Fig. S1a, b). Patients were all HBV-infected except for one HCV-infected (P05) with 3 cases at stage I and 4 cases at stage III (Supplementary Table S1). A total of 41,698 cells passed quality control, including 21,991 from non-tumor liver tissues (Fig. 1d, e) and 19,707 cells from HCC (Fig. 1f, g), with the mean number of 5549 UMIs/cell and 1709 genes/cell (Supplementary Table S2). We used the Single R algorithm^15^ to group cells into main immune cell types and visualized by t-Distributed Stochastic Neighbor Embedding (t-SNE). We identified eight immune subsets with the expression of well-known marker genes (Fig. 1h). Consistent with previous data^12^, T cells were the most abundant type of immune cells in tumor (36.20%), followed by NK (28.57%) and macrophages (25.04%). Although the number of dendritic cells (DC) detected was the least (1.19%), the number of UMIs and genes sequenced in them was the most (16,438 UMIs/cell and 3187 genes/cell; Supplementary Fig. S1c–f).

### Myeloid cells are functionally diversified in HCC microenvironment

**Fig. 2:**
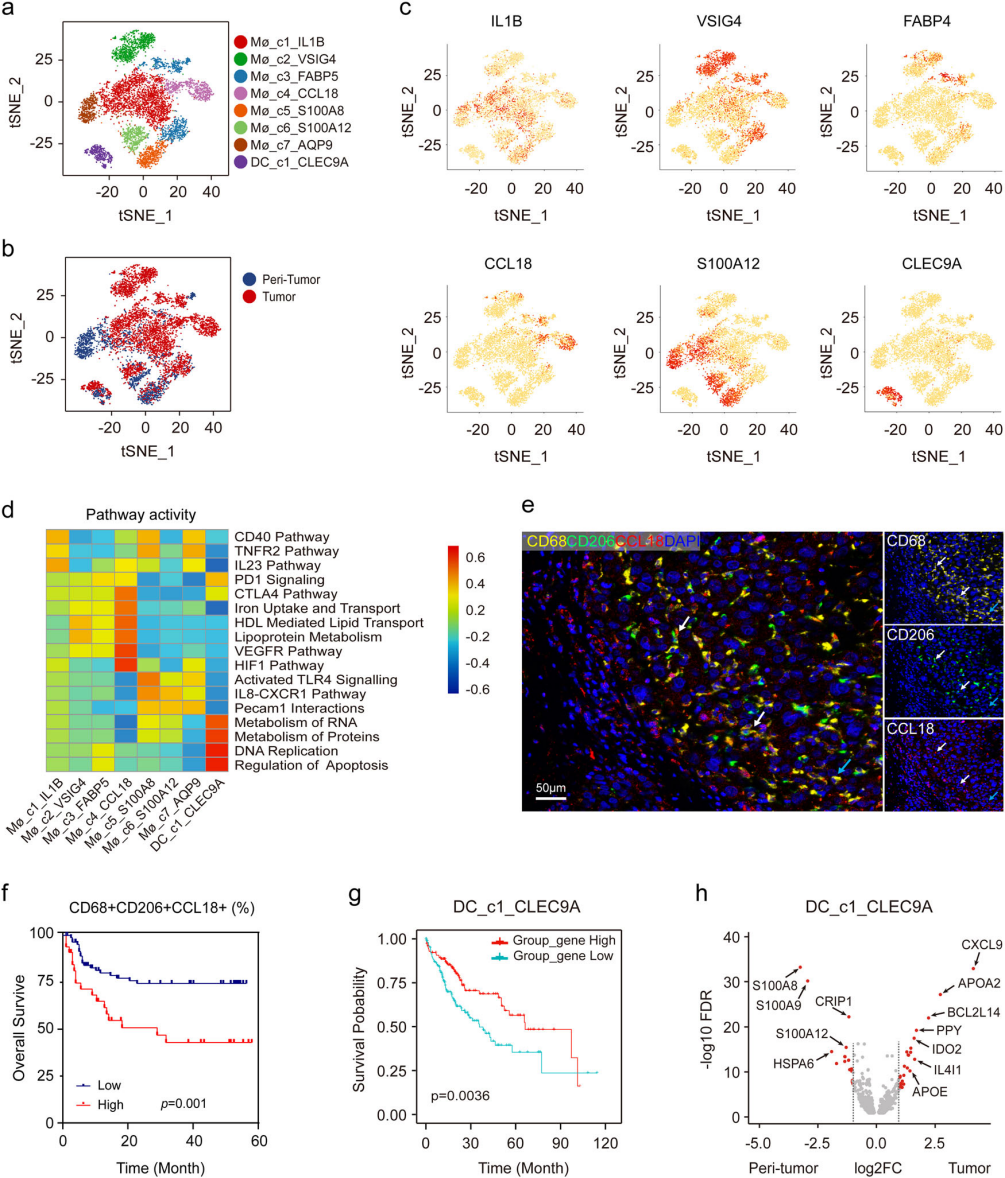
Identifying distinct myeloid cell clusters in HCC. **a** t-SNE projection of eight subsets of myeloid cells (each dot corresponds to one single cell) shown in different colors. **b** t-SNE plots of different myeloid cell clusters origin. **c** Expression of marker genes for each cluster illustrated in the t-SNE plots. **d** Heatmap of the differences in pathway activities scored per cell by GSVA analysis. **e** Representative mIHC images to show the distribution of CD68^+^CD206^+^CCL18^+^ macrophages: CD68 (yellow), CD206 (green), CCL18 (red), and DAPI (bule). White arrows (CD68^+^CD206^+^CCL18^+^), blue arrow (CD68^+^CD206^+^CCL18^−^). Scale bar, 50 μm. **f** Kaplan–Meier curve showing poor survival in patients with high proportion of CD68^+^CD206^+^CCL18^+^ macrophage vs low proportion (log-rank test, *P* = 0.001) in our cohort. **g** Kaplan–Meier curves of survival for the TCGA HCC patients grouped by the average expression (high versus low) of DC_c1_CLEC9A cell marker genes as annotated in Table S3. (log-rank test, *P* = 0.0036). **h** Volcano plot showing differentially expressed genes in DC_c1_CLEC9A cells between peri-tumor and tumor. Each red dot denotes an individual gene passing our *P* value and fold change thresholds.

Myeloid cells consist of various subsets and exhibit distinct functions in tumor immunity. To further investigate myeloid populations in HCC, unsupervised clustering 7008 myeloid cells clearly revealed eight distinct clusters (Fig. 2a, b; Supplementary Fig. S2a–e).

In our data, Mø_c1 represented the most abundant macrophages (37.10%) with high expression of *IL1B*, as well as *CXCL10* and *CXCL9* (Fig. 2c; Supplementary Table S3), which might be involved in anti-tumor responses^16^. Consistently, these macrophages expressed higher levels of IFN-γ related genes such as *TNFAIP3*, *GBP1*, *APOBEC3A*, and *GBP5*. Mø_c2 and Mø_c3 (13.54% and 13.31%, respectively) were considered as Kupffer-like cells, owing to their higher expression of *VSIG4*, a membrane protein specific to tissue-resident macrophages^17^. Remarkably, Mø_c4 (11.16%) was mostly infiltrated in advanced HCC patients (P04 and P07), and characterized by higher expression of *CCL18*, which was absent in previous scRNA-seq study^12^. These macrophages showed strong activity in lipid transport and metabolism, and immunosuppressive-related pathways (Fig. 2d). We confirmed that CCL18 was mainly secreted by M2 macrophages (Fig. 2e) and HCC patients displayed a higher proportion of CD68^+^CD206^+^CCL18^+^ macrophages in tumor significantly associated with large tumor size (*P* = 0.025), advanced TNM stage (*P* = 0.034; Supplementary Table S4), and poor survival (Fig. 2f, *P* = 0.001; Supplementary Fig. S2f) in our cohort.

Mø_c5-Mø_c7 showed a strong donor phenotype and were defined as monocytic myeloid-derived suppressor cells (M-MDSCs, 8.78%, 8.16%, and 7.95%, respectively), characterized by the high expression of *S100A12*, *S100A9*, and *S100A8*^18^ (Supplementary Fig. S2g), as well as *CCR2*, which could facilitate their trafficking to tumor site^19^. In particular, Mø_c7, mostly derived from P06, a 36 years male patient with a high load of HBV DNA (127,000 IU/mL), showed upregulation of *IFI44L* and *IFI6*, implicating a potential role in antiviral responses^20,21^.

We only recovered one subset of DC (DC_c1) with enriched expression of *CLEC9A* and *XCR1*, markers of antigen-presenting cDC1 cells, partly due to the sample bias or different tissue digestion methods. cDC1 cells play an important role in activating T cells by presenting antigens^22^. As expected, HCC with increased DC_c1 marker gene expression had significantly better survival in TCGA cohort (*P* = 0.0036, Fig. 2g). Interestingly, DC_c1 showed enrichment in pathways related to RNA and protein metabolism and DNA replication, implying their upregulated transcription, consistent with the findings that they possessed the highest number of genes. Furthermore, DC_c1 cells might have functional alteration and enhanced lipid utilization due to the remodeling of TME as the increased expression of *CXCL9, IDO2, APOA2*, and *APOE* compared to their non-tumor counterparts (Fig. 2h).

### Transcriptome heterogeneity of different subsets of macrophages

**Fig. 3:**
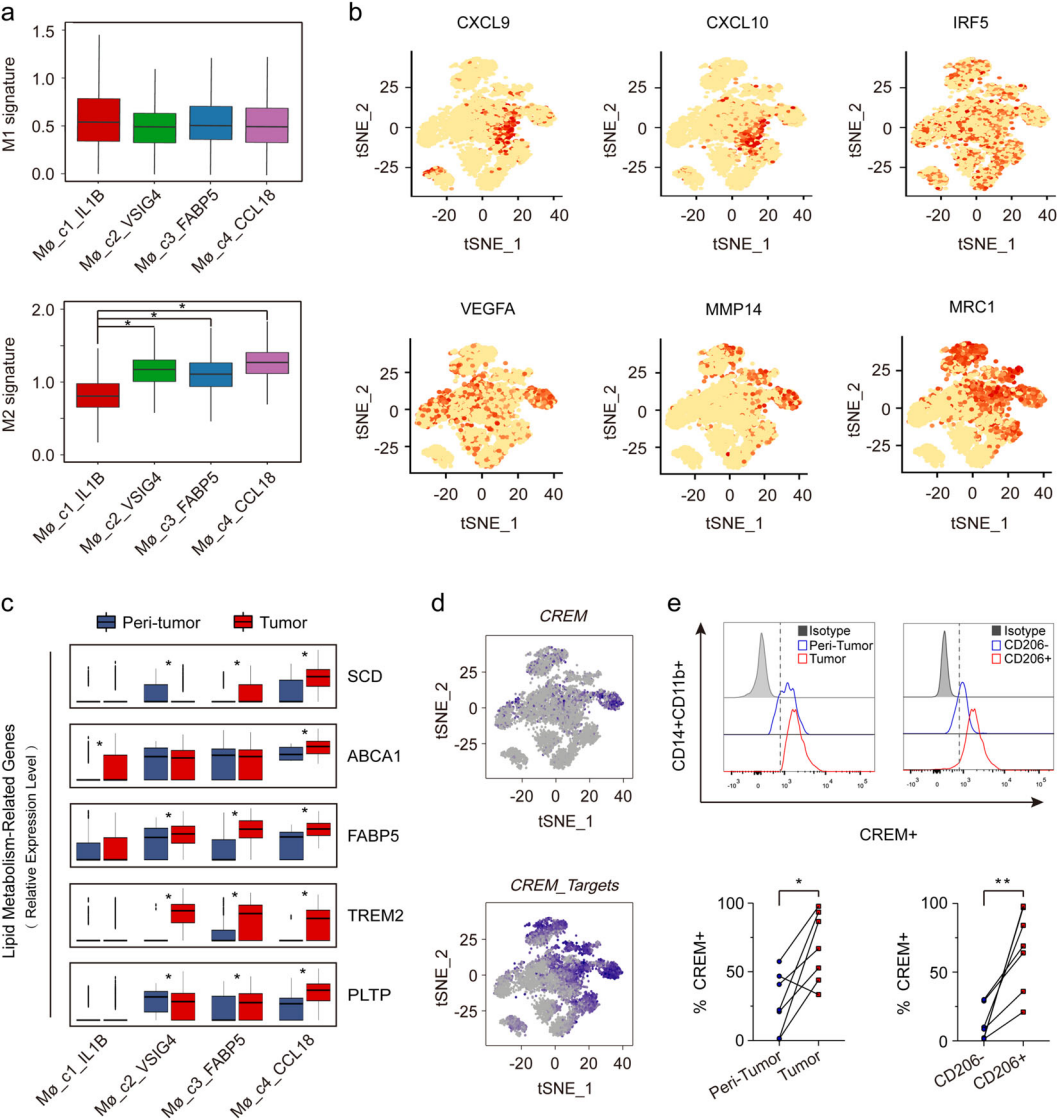
Transcriptome heterogeneity of four subsets of macrophages. **a** Module scores of M1 and M2 expression signatures defined by Azizi et al.^14^ (Genes list in Supplementary Table S5) for each macrophage subset at single-cell level. *P < 0.01. **b** t-SNE plots of M1 (top) and M2 (bottom) expression signatures. **c** The expression of lipid metabolism-related genes plotted via boxplots. *P < 0.01. **d** t-SNE plots for the expression of *CREM* and regulation of its target genes. **e** Representative flow cytometry plots (top) and statistics (bottom) of CREM expression in CD14^+^CD11b^+^ macrophages from HCC tumor or peri-tumor, and CD14^+^CD11b^+^CD206^−^ or CD14^+^CD11b^+^CD206^+^ macrophages. Data analyzed by wilcoxon matched-pairs signed rank test. *P < 0.05, **P < 0.01.

Macrophages are phenotypically and functionally plastic, but the model of macrophage polarization remains controversial. We assessed the expression of M1 and M2 signature genes^14^ (Supplementary Table S5) in Mø_c1-Mø_c4 to define their phenotypes and performed Monocle 2 algorithm^23^ to reveal their potential transition (Supplementary Fig. S3a). Results showed that Mø_c1 and Mø_c4 were phenotypically more like M1 and M2 macrophages respectively, while Mø_c2 and Mø_c3 appeared at intermediate stages (Fig. 3a). Along the transition from M1 to M2 state, macrophages acquired features that promote tumor invasion, metastasis, and immunosuppression with upregulated genes like *MMP14*, *VEGFA*, and *MRC1* (Fig. 3b; Supplementary Fig. S3b). However, although macrophages gradually obtained the characteristics of M2 phenotype, they did not obviously down-regulate M1 signature. This finding indicated that M2 like macrophages still maintained some anti-tumor properties, supporting the view that macrophage activation in TME did not follow the classical polarization pattern^14,24^.

Macrophage may alter the metabolic gene expression to accommodate energy requirements, such as increased fatty acid oxidation providing a crucial energy source for M2 polarization^25^. We found that M2 macrophages showed progressively enhanced lipid metabolism compared with M1 macrophages (Fig. 3c), as exemplified by highly expressed *TREM2* in Mø_c2-Mø_c4, which is a marker gene of M2 macrophages^26^. Moreover, M2 macrophages in tumors exhibit stronger lipid metabolism characteristics than those in non-tumors, indicating that TME might enhance lipid metabolism in M2 macrophages. Lipid metabolism-related genes *FABP5*, *ABCA1*, *SCD*, and *PLTTP* were heterogeneously expressed in M2 macrophage subsets, indicating metabolic heterogeneity among different M2 subsets.

Focusing on the M2 closest macrophages in Mø_c4, we found the transcription factor *CREM* was expressed more strongly in this cluster, and its target genes were also highly upregulated in Mø_c2-Mø_c4 macrophages by Single-Cell Regulatory Network Inference and Clustering (SCENIC)^27^ analysis (Fig. 3d; Supplementary Fig. S3c, d). CREM is capable of binding to *IL2* promoter to decrease its production in T cells^28^, however, whether *CREM* is expressed in macrophages remains unclear. We further confirmed that *CREM* significantly upregulated in M2 macrophages in HCC by flow cytometry (Fig. 3e), whose exact role needs functional investigation.

### Immunomodulatory and cytotoxic effects of diverse status of NK cells

NK cells are phenotypically defined as CD56^bright^ and CD56^dim^, which play different roles in TME. Recently, several new subsets of NK cells have been identified by scRNA-seq in blood and spleen from non-neoplastic patients^29–31^, implying the tissue-related diversity of NK cells.

**Fig. 4:**
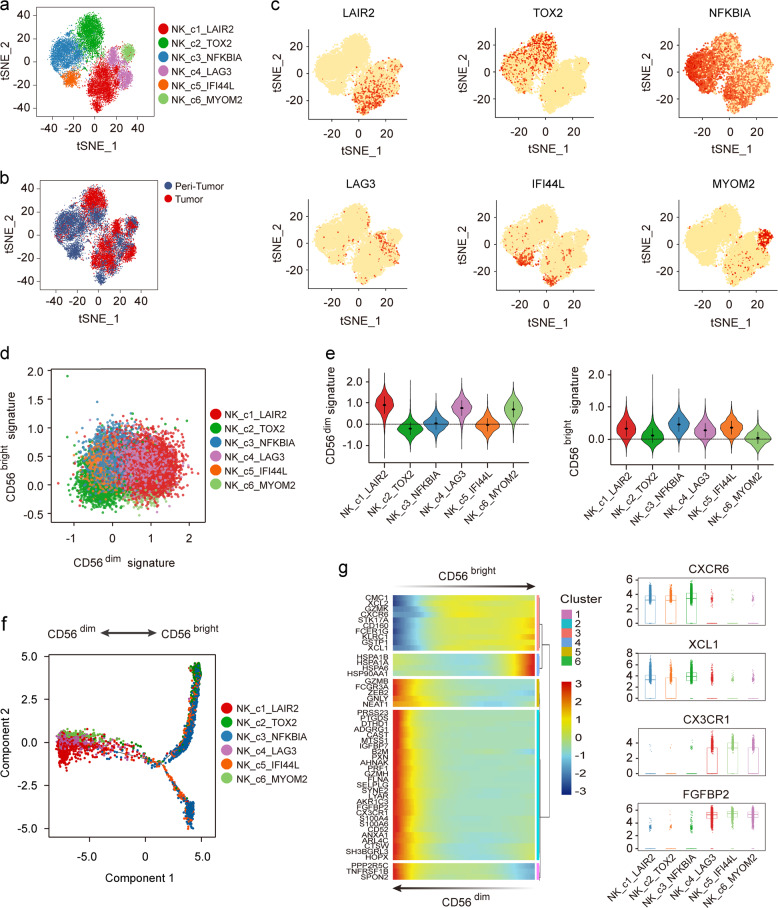
Different NK subpopulations in HCC tumor and peri-tumor. **a** t-SNE plot of all NK cells revealed six distinct NK clusters. **b** t-SNE plots of different NK cell clusters origin. **c** Expression of canonical marker genes in six NK cell populations. **d** Module scores of CD56^bright^ and CD56^dim^ expression programs defined by Hanna et al.^33^ for each NK cell. **e** Violin plots representing the distribution module score for CD56^dim^ (left) and CD56^bright^ (right) for each NK cluster. Error bars indicated the means ± SD. **f** Trajectory of all clusters of NK cell from tumor sites along pseudotime in a two-dimensional state-space defined by Monocle2. Each point corresponds to a single cell, and each color represents a NK cell cluster. **g** Differentially expressed genes (rows) along the pseudotime (left) and boxplots showing the expression of *CXCR6*, *XCL1*, *CX3CR1*, and *FGFBP2* (right).

We identified six subsets of NK cells (14,934 cells) by unsupervised clustering (Fig. 4a, b; Supplementary Fig. S4a–d). Immune subsets of NK cells were highly donor specific, which may reflect differences in genetic origin or adaptability to different TMEs among individuals. NK_c3 and NK_c5 were mainly derived from non-tumor liver tissues (23.57% and 5.97%, respectively) and characterized by high expression of transcription factors such as *FOS*, *FOSB*, *FOXP1*, and *ATF4*, and two genes involved in the NF-κB pathway, *NFKBIA* and *NFKBIZ* (Fig. 4c; Supplementary Fig. S4e). Moreover, NK_c5, mostly derived from P06 with high load of HBV DNA as mentioned before, may exert additional antiviral effect because of their specific expression of antiviral related genes like *IFI44*, *IFI44L*, and *MX1* (Supplementary Table S3), which has not been revealed by non-HBV populations^29^. Consistently, NK_c5 highly expressed *STAT2*, *IRF9*, and *IRF7* (Supplementary Fig. S4f), which contribute to the transcriptional activation of multiple virus-inducible genes^32^. Additionally, we also recovered one terminal NK subset (NK_c1, marker genes: *LAIR2*, *IGFBP7*, and *CD55*, 27.66%), one exhausted NK subset (NK_c4, marker genes: *LAG3*, *PTMS*, and *S100A6*, 13.55%), and two undefined NK subsets (NK_c2, marker genes: *TOX2*, *CXCR6*, and *XCL1*, 23.81%, and NK_c6, marker genes: *MYOM2*, *CX3CR1*, and *PRF1*, 5.60%).

We found NK cells mainly formed two distinct subgroups as NK_c1, NK_c4, and NK_c6 strongly expressed cytotoxicity related genes like *GZMB*, *GNLY*, *FGFBP2*, and *FCGR3A* (CD16), while NK_c2, NK_c3, and NK_c5 expressed higher levels of *GZMK*, *CXCR6*, and *CD69*, marker genes of CD56^bright^ tissue-resident NK cells (Supplementary Fig. S4g). We then compared the expression profiles of these NK subsets with CD56^bright^ and CD56^dim^ gene expression programs to clarify their properties defined by Hanna et al.^33^ (Fig. 4d). Results showed that NK_c1, NK_c4, and NK_c6 were more like CD56^dim^ NK cells (Fig. 4e), and FCM confirmed the higher expression of Granulysin, Granzyme B, KIR2DL1, and CX3CR1 in these NK cells (Supplementary Fig. S4h), suggesting their strong cytotoxic functions. Interestingly, NK_c1 and NK_c4 also scored higher points in CD56^bright^ signature like NK_c3 and NK_c5, implying the immunoregulation effects of NK cells was not limited to CD56^bright^ cells and conventional classification of NK cells was not suitable for all NK subsets.

We also identified the continuous process of CD56^dim^ NK cells transformed from CD56^bright^ NK cells (Fig. 4f), supporting the notion that CD56^bright^ NK cells are the precursors of CD56^dim^ NK cells^30^. CD56^bright^ NK cells expressed higher levels of membrane receptors such as *CXCR6*, *CD160*, and *KLRC1*, as well as chemokines like *XCL1* and *XCL2*, indicating their immuno-regulatory effects in TME. Expression of membrane receptors such as *FCGR3A* and *CX3CR1*, and cytotoxic genes like *GZMB*, *FGFBP2*, *PRF1*, and *GNLY* increased gradually during this transition, implying a gradually acquired tumor-killing ability in CD56^dim^ NK cells (Fig. 4g).

### Infiltration of XCL1^+^CD8^+^ T cells indicates a better prognosis for HCC patients

**Fig. 5:**
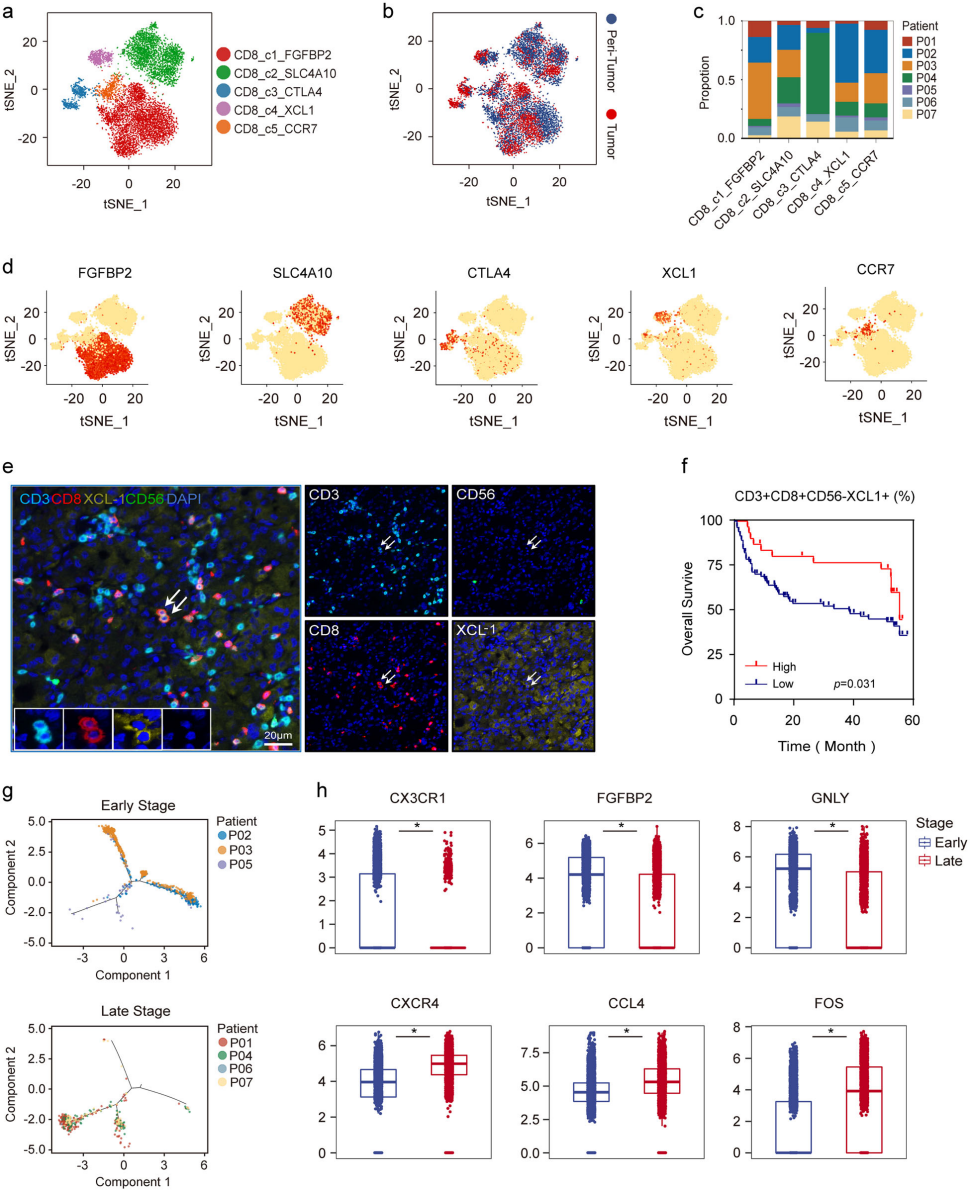
Infiltration of diverse CD8^+^ T-cell subsets in HCC. **a** t-SNE projection of all CD8^+^ T cells showed in different colors. **b** t-SNE plots of different subsets of CD8^+^ T-cell origin. **c** Proportions of five clusters in each patient. **d** Expression of marker genes for each cluster illustrated in the t-SNE plots. **e** Multicolor IHC staining to validate the existence of CD3^+^CD8^+^CD56^−^XCL1^+^ T cells in HCC TME, white arrows (CD3^+^CD8^+^CD56^−^XCL1^+^). Scale bar, 20 μm. **f** Kaplan–Meier curve showing decreased survival in patients with low proportion of CD3^+^CD8^+^CD56^−^XCL1^+^ T cells (Log-rank test) in our cohort (Supplementary Table S4). **g** Pseudotime trajectory of early-stage or late-stage CD8_c1 T cells demonstrated in the trajectory. **h** Expression of selected genes in early-stage and late-stage HCC are shown in the boxplots. *P < 0.01.

We detected 8487 CD8^+^ T cells and re-clustered to five subsets (Fig. 5a–d; Supplementary Fig. S5a–c), including effector CD8^+^ T cells (CD8_c1, marker genes: *FGFBP2*, *GZMB*, and *GNLY*, 48.17%), MAIT cells (CD8_c2, marker genes: *SLC4A10*, *KLRB1*, and *ZBTB16*, 31.91%), exhausted CD8^+^ T cells (CD8_c3, marker genes: *CTLA4*, *HAVCR2*, and *PDCD1*, 7.51%), activated XCL1^+^CD8^+^ T cells (CD8_c4, marker genes: *XCL1*, *XCL2*, and *ITGAX*, 6.19%) and central memory CD8^+^ T cells (Tcm, CD8_c5, marker genes: *CCR7*, *SELL*, and *GPR183*, 5.34%).

We focused our analysis on the CD8_c4, XCL1^+^CD8^+^ T cells, as this cluster has not been characterized by previous scRNA-seq studies in HCC^12,13^. It had been reported that CD8^+^ T cells could secret XCL1 when they were activated, and then recruited XCR1^+^ DC cells for antigen presentation^34^. Data showed that CD8_c4 T cells expressed high levels of memory markers like *ITGA1* and *CD7*, and genes for the recognition of MHC class I molecules such as *KLRC2*, *KIR2DL3*, and *KIR3DL2* (Supplementary Table S3), implying their important roles in antigen recognition and immune activation. Recently, *XCL1* showed upregulation in subset of tissue-resident memory CD8^+^ T cells (C5_CD8-GZMK) and exhibited a possible “pre-exhaustion” state by previous scRNA-seq study in HCC^13^. However, in our data, XCL1^+^CD8^+^ T cells formed a subset that different from CD8^+^GZMK^+^ T cells because of their more specific *XCL1* expression and relatively low *GZMK* expression (Supplementary Fig. S5d). We examined the expression of *XCL1* in this subset by multiplex IHC (mIHC), excluding NK cells as they could also secrete XCL1 (Supplementary Fig. S5e). In our cohort, we confirmed the presence of one subset of CD3^+^CD8^+^ T cells which abundantly secreted XCL1 (Fig. 5e), and patients with a higher density of these cells, rather than NK cells, had a better prognosis (Fig. 5f, *P* = 0.031; Supplementary Fig. S5f).

We next evaluated the developmental course of CD8^+^ T cells (Supplementary Fig. S6a, CD8_c2, MAIT cells were removed as their different origins). Cells of CD8_c1 (effector CD8^+^ T cells) and CD8_c3 (exhausted CD8^+^ T cells) located at the opposite ends of the trajectory, whereas cells of activated and Tcm CD8^+^ T cells of CD8_c4 and CD8_c5 located in the middle part. Moreover, effector CD8^+^ T cells demonstrated more branches than other three types, suggesting their greater heterogeneity in anti-tumor responses. We investigated genes whose expression levels gradually increased with exhaustion. In addition to the known exhaustion markers such as *CTLA4*, *PDCD1*, *TIGIT*, *HAVCR2*, and *LAG3*, several target genes that could potentially promote exhaustion of CD8^+^ T cells, such as FABP5, TRPS1, CREM, and CEBPD were also discovered in our study (Supplementary Fig. S6b, c).

### Functional impairment of effector CD8^+^ T cells in advanced HCC

Overall, patients in early-stage HCC (P02, P03, and P05) had a higher proportion of effector CD8^+^ T cells (CD8_c1), while patients in late-stage HCC (P01, P04, P06, and P07) contained a higher proportion of exhausted CD8^+^ T cells (CD8_c3) (Supplementary Fig. S7a). So, we speculated that the effector CD8^+^ T cells of patients in early-stage may transcriptionally differ from those in advanced HCC due to the long-term remodeling of the TME. A pseudotime map of effector CD8^+^ T cells in tumors was constructed to reflect the transcriptomic changes among different patients. Cells from early-stage HCC were mainly distributed at one side of the trajectory, while cells from advanced HCC mainly located at the other side (Fig. 5g; Supplementary Fig. S7b), suggesting distinct expression profiles between early-stage and advanced HCC. Then, genes that changed dramatically along the trajectory were analyzed. Interestingly, genes involved in CD8^+^ T-cell cytotoxicity such as *CX3CR1*, *FGFBP2*, *GNLY*, and *NKG7* were down-regulated in advanced HCC, indicating their damaged cytotoxicity (*P* < 2.2 × 10^–16^). In contrast, *CXCR4*, which has been shown to be inversely related to the expression of perforin and its blockage was shown to activate the migration and tumor-killing ability of CD8^+^ T cells^35,36^ was found upregulated in advanced HCC. Also, cellular stress response related genes such as *FOS*, *JUND*, *JUNB*, and *JUN* showed significant upregulation in advanced HCC (*P* < 2.2 × 10^–16^), implying these cells might be engaged in complex transcriptional reprogramming probably due to the disruption of the TME (Fig. 5h; Supplementary Fig. S7c and Table S6). Together, our results indicated a damaged function of effector CD8^+^ T cells in advanced HCC that may lead to impaired anti-tumor response.

### Distinct subsets of CD4^+^ T cells and B cells identified in HCC

We identified five clusters of CD4^+^ T cells by analyzing 7533 CD4^+^ T cells (Supplementary Fig. S8a–g), including central memory CD4^+^ T cell (CD4_c1, marker genes: *CCR7*, *TCF7*, and *IL7R*, 39.19%), Treg cells (CD4_c2, marker genes: *FOXP3*, *TIGIT*, and *CTLA4*, 24.64%), T helper cells (CD4_c3, marker genes: *CCL5, GZMK*, and *GZMA*, 22.55%), cytotoxic like CD4^+^ T cells (CD4_c4, marker genes: *NKG7*, *FGFBP2*, and *GNLY*, 13.59%), and a small amount of antiviral-related CD4^+^ T cells (CD4_c5, marker genes: *IFIT1*, *IFIT2*, and *IFIT3*, 0.72%). The SCENIC analysis showed that genes regulated by *MYC* were specifically upregulated in CD4_c1. *MYC* plays an important role in cell cycle progression and can upregulate the transcription of various target genes. Pathway analysis supported this phenomenon as the significantly increased pathways were involved in nucleotide metabolism of proteins and RNAs (Supplementary Fig. S8h, i).

We also recovered five subsets of B cells (3240 cells, Supplementary Fig. S9a–g), including memory B cells (B_c1, marker gene: *AIM2* and *GPR183*, 48.02%), unswitched naïve B cells (B_c2, marker genes: *TCL1A*, *IGHD*, and *IL4R*, 24.38%), plasma cells (B_c3, marker genes: *XBP1*, *MZB1*, and *CD38*, 13.86%), tumor-specific B cells (B_c4, marker genes: *APOA2*, *APOC1*, and *APOA1*, 8.58%), and atypical memory B cells (B_c5, marker genes: *SLC11A1*, *FGR*, and *TNFRSF1B*, 5.15%).

### Intercellular communication of diverse immune subsets in microenvironment

**Fig. 6:**
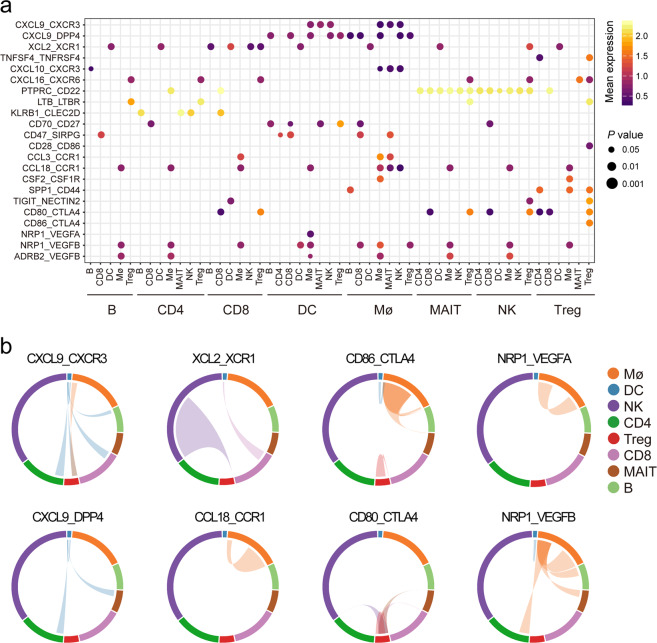
Increased cell–cell interactions occurring in the HCC TME. **a** Overview of selected ligand–receptor interactions which presented specifically in HCC tumors. *P* values indicated by circle size (permutation test). The means of the average expression level of interactions are indicated by color. The cell types below the line are the ligand cells, and the cell types above the line are the corresponding receptor cells. **b** Selected interactions of ligand-receptor pairs in HCC TME. The line color indicates ligands broadcast by the cell population of the same color. Lines connect to cell populations where cognate receptors are expressed. The line thickness is proportional to the number of ligands where cognate receptors are present in the recipient cell population.

Cell–cell interactions in TME are critical for tumorigenesis and progression. We performed CellphoneDB^37^ analysis to clarify interactions between immune subsets and identified interactions specific for tumors (Fig. 6a; Supplementary Table S7). Results showed that interactions participating in immune activation and anti-tumor response, such as, *CXCL9-CXCR3*, *CXCL9-DPP4*, *XCL2-XCR1*, and *TNFSF4-TNFRSF4* upregulated in tumors, partly due to the continuous stimulation of tumor antigens. Interestingly, we found that various immunosuppressive interactions were also upregulated in tumors. For example, CCR1^+^ monocytes had been revealed to facilitate immune escape in HCC by our previous study^38^, here we also found that macrophage-derived *CCL18* can form self-feedback with receptor *CCR1*, potentially promoting tumor immunosuppression (Fig. 6b). Also, interactions such as *CD80-CTLA4*, *CD86-CTLA4*, and *LGALS9-HAVCR2*, were mostly enriched in tumor, implying that these interactions might jointly conduce to immune escape. Moreover, interactions related to tumor angiogenesis, such as *NRP1-VEGFB*, *ADRB2-VEGFB*, and *NRP1-VEGFA* were extensively recovered in tumor, implying their potential roles in promoting tumor angiogenesis.

## Discussion

The TME has profound impacts on immunotherapeutic response and clinical outcome. Although previous scRNA-seq studies in HCC have revealed new T-cell subsets and immune cell migration among different tissues^12,13^, the immune subset landscape within HCC, which is easily affected by data analysis, is far from full interpretation. We here uncovered a high degree of spatiotemporal heterogeneity in the distribution, functional properties, transcriptional regulation, and cell–cell interactions of different immune subsets in HBV/HCV-related HCC. We also identified two new subsets, including CCL18^+^ M2 macrophages enriched in advanced HCC, and XCL1^+^ CD8^+^ T cells capable of recruiting DC to enhance anti-tumor response. Our study depicted a global map of immune cell subsets in the liver and represented an important basis for understanding the immune regulation in HBV/HCV-related HCC.

Macrophages display noticeable plasticity in phenotypic and functional properties. We uncovered four subsets of macrophages that gradually transitioned from M1 to M2 phenotype. Consistent with previous reports^14^, our data showed that M2 macrophages still retain some characteristics of M1 macrophages, in spite of their obvious tumor-promoting functions, indicating the dual role of M2 macrophages in anti-tumor responses. Unexpectedly, we observed significant heterogeneity and upregulation of lipid metabolism during the transition of M1 to M2 states. It is possible that the extreme conditions inside the tumor, such as hypoxia, can cause aberrant expression of certain genes, such as HIF1α, leading to metabolic reprogramming^39^. We especially observed one M2 subpopulation that was featured by high expression of CCL18 and mainly presented in advanced HCC. These macrophages showed well-defined features that promote tumor invasion, angiogenesis, metastasis, and was associated with poor prognosis in HCC. To date, CCL18^+^ macrophages have been linked to various cancers such as breast^40^ and gallbladder^41^, suggesting the presence of such macrophages is a common feature in cancer and may be a therapeutic target. Moreover, we identified transcription factor CREM and its putative target genes especially upregulated in this subset. Although the precise regulatory mechanism of CREM in CCL18^+^ M2 macrophage remains poorly understood, in vivo study has shown that tumor acidosis can induce CREM expression in macrophages, promoting their non-inflammatory polarization^42^. Thus, a likely essential function of CREM in M2 macrophages is to mediate immunosuppression, which may be a potential target for tumor immunotherapy, but the mechanistic details need further study.

We identified one subset of CD8^+^ T cells with high secretion of XCL1 that correlated with better prognosis. XCL1 is mainly produced by activated NK cells and CD8^+^ T cells, and considered to be the only ligand of receptor XCR1which is selectively expressed in cDC1 cells. Our data showed that XCL1 mainly expressed in a subpopulation of CD8^+^ T cells that characterized as a possible “pre-exhaustion” status in HCC^13^. These cells play an active role in anti-tumor effects partly by secreting XCL1 and recruiting cDC1 cells for tumor antigens presenting, which will, in turn, attract more CD8^+^ T cells to exert cytotoxic response. Of note, a recent study has shown that XCL1 is abundantly secreted by antigen-responsive CD8^+^ T cells^43^, which further indicates the initial activation status of these T cells and facilitates us to map the antigen-specific T-cell receptors (TCR) sequence. Together, our findings highlight the importance of XCL1-XCR1 axis in anti-tumor response, indicating that immunotherapeutic effect may be enhanced by recruiting XCL1^+^CD8^+^ T cells into HCC.

We observed a continuous evolution in cytotoxic CD8^+^ T cells of HCC at different stages. Our results showed that early-stage HCC had a higher proportion of cytotoxic CD8^+^ T cells and displayed strong cytotoxicity, while advanced HCC showed an increase proportion of exhausted CD8^+^ T cells and decrease proportion of cytotoxic CD8^+^ T cells with weakened killing ability. This is partly due to the chronic stimulation of tumor antigens and continuous remodeling of TME, which may eventually alter the phenotype and function of immune subsets. Furthermore, since advanced HCC has more infiltration of M2 macrophages and Treg cells, it will further dampen the anti-tumor ability of cytotoxic CD8^+^ T cells^44^.

Several limitations of this study need to be considered. First, the number of patients in our study is relatively small (*n* = 7), and these patients are either HBV- or HCV- related HCC. Therefore, these results and conclusions may not be applied to patients with fatty liver or alcohol related liver cancer. Second, there were relatively significant individual differences in AFP level, virus antigen, HBV DNA copies, and liver cirrhosis among HCC patients in our cohort, which may affect the composition, transcriptional profile, phenotype and function of immune cells, as well as the presence of donor specific immune subsets (especially in myeloid and NK subsets). Future work will need to be performed in a larger cohort to validate these identified immune subsets and explore their specific role in HCC.

In summary, our study delineates the landscape of diverse immune subsets and their underlying transcriptome dynamics during tumor progression. This comprehensive analysis extends our understanding of the role of multiple immune subsets in HBV/HCV-related HCC and may also contribute to the development of new therapeutic targets and strategies.

## Materials and methods

### Patient samples

Seven patients had liver resection and were pathologically diagnosed HCC in January 2019 were enrolled for scRNA-seq. None of the patients had received anti-tumor treatments before surgery. Fresh tumor tissues and distant non-malignant liver were obtained from each patient. This study was conducted in accordance with the ethical standards of the Research Ethics Committee of Zhongshan Hospital with patients’ informed consent.

### Tissue microarray

Tissue microarrays (TMA) were produced as described previously^45^. All HCC cases (*n* = 121) who underwent primary resection between January and April 2008, were histologically reviewed by H&E staining and representative areas were pre-marked in the paraffin blocks, away from necrotic and hemorrhagic materials. Sections of 4 μm thick were placed on slides coated with 3-aminopropyltriehoxysilane. None of the patients received anti-tumor or immunosuppressive treatments before surgery.

### Preparation of single-cell suspensions

After resection, fresh tumor and adjacent non-malignant liver tissues were transferred rapidly to the 50 mL centrifugal tube filled with DMEM (Gibco) medium with 10% fetal bovine serum (Gibco) and transported rapidly to the laboratory on ice. On arrival, samples were transferred to a 6-cm dish and washed twice with 1× cold PBS (Gibco). Each sample was cut into ~1 mm^3^ piece on ice and was subsequently transfered into 10 mL digestion medium containing 1 mg/mL collagenase IV (Gibcol, 17104019) and 1 U/mL dispase II (Gibcol, 17105041). Samples were incubated at 37 °C for 40 min and stirred every 10 min with a pipette tip. The dissociated cells were subsequently passed through a 40-µm cell-strainer nylon mesh (BD) and centrifuged at 700 × *g* for 10 min. After centrifugation, the supernatant was removed, and the cell pellet was washed twice with MACS buffer (PBS containing 1% FBS, 0.5% EDTA, and 0.05% gentamycin) and then re-suspended in sorting buffer (PBS supplemented with 1% FBS). To maximize more cell types, cells were only stained with DRAQ5 (1:200, 15 min) and DAPI (1:200, 15 min) to harvest nucleated living cells. Stained cells were then run on MoFlo Astros EQ (Beckman Coulter) Cell Sorter and sorted into DMEM media supplemented with 10% FBS.

### Single-cell RNA-sequencing

Chromium Single Cell 3′ Reagent Kits (V3) were used to prepare individually barcoded single-cell RNA-seq libraries following the manufacture’s protocol (10× Genomics). The isolated single cells were loaded in each channel with a target output of 6000 cells per sample. For sequencing library construction, single-cell suspensions were loaded on a 10× Genomics Single-Cell Instrument and were partitioned in droplets. GEM-RT was performed: 53 °C for 45 min, 85 °C for 5 min. Sequencing was conducted on Illumina Sequencer (NovaSeq). The Cell Ranger Single-Cell SoftwarTABLEe suite was used for demultiplexing, barcode processing, alignment, and initial clustering of the raw scRNA-seq profiles. Raw sequencing reads were mapped to the human genome (build GRCh38, ENSEMBL), annotated and quantified based on the GRCh38 reference annotation file (ENSEMBL) using Cellranger pipeline (v3.0.1). Cells that had fewer than 2000 UMIs, or 800 genes, or more than 20% UMIs mapped to the mitochondrial genome were filtered. To remove possible doublets, we used Scrublet (v0.1), Doublet Finder (v2.0) and Doublet Detection (v2.4) together and removed those cells labeled as doublets by more than two software. Moreover, we manually examined the expression of classical markers and removed the cells expressing conflicting markers.

### Determination of cell type and clustering

Cells were classified into the major cell types in the HPCA database using SingleR^15^ (v1.0). Five major cell types (Myeloid cell, NK cell, CD8^+^ T cell, CD4^+^ T cell, and B cell) remained after excluding those cell types fewer than 100 cells. Seurat (v2.3.4) was used for downstream analysis. For each cell type, gene expression matrices were normalized to cell library size and log-transformed using Scale Data function. We assigned cell cycle score (G1/S or G2/M) for each cell and found there were fewer than 3% cells were mitotic cells. Thus, we did not correct our data for the effects of the cell cycle. After normalization, we identified most variably expressed genes using Find Variable Genes function and performed principal component analysis by Run PCA function. The first 30 principal components were selected for clustering using Find Clusters function. The marker genes for each cluster was determined using Find All Markers function.

### Differential expression and pathway analysis

Differentially expressed genes (fold change > 4 and *P* value < 0.001) were identified using the QLF model implemented in edgeR (a Bioconductor package for differential expression analysis of digital gene expression data, v3.26.3) after correcting for the patient of origin. Gene set variation analysis was performed using the gene set variation analysis for microarray and RNA-Seq data (GSVA, v1.32.0) and the gene sets (Hallmark pathways, Canonical pathways) were derived from MSigDB (http://software.broadinstitute.org/gsea/msigdb).

### Multiplex immunohistochemistry and quantitative analysis

Multiplex immunohistochemistry (mIHC) was performed according to manufacturer’s instruction (PerkinElmer, Opal^®^ Kit). Slides were scanned and imaged using the PerkinElmer Vectra3^®^ platform and were analyzed in batches using PerkinElmer inform and R script for quantification of positively stained cells. The primary antibodies and dilutions used were listed in CTAT_table. Briefly, TMA sections were de-paraffinized in xylene and rehydrated in ethanol. After microwave antigen retrieval in heated citric acid buffer (pH 6.0) for 10 mins, endogenous peroxidase activity was blocked by 3% H_2_O_2_ for 30 mins, and nonspecific binding sites were blocked by goat serum (Vector,11-06-18) for 30 mins. Primary antibodies were incubated for 1 h in a humidified chamber at room temperature, followed by corresponding secondary horseradish peroxidase-conjugated polymer. Visualization of each target was accomplished using fluorescein TSA Plus (1:200). Then the slide was again placed in heated citric acid buffer (pH 6.0) using microwave antigen retrieval to remove redundant antibodies before the next step. Finally, nucleis were subsequently visualized with DAPI (Sigma, D9542), and the section was coverslipped using fluorescence mounting media (DAKO, S3023). Slides were scanned and imaged using the PerkinElmer Vectra3^®^ platform and were analyzed in batches using PerkinElmer inform and R script for quantification of positively stained cells. The cut-off values of proportions of CD68^+^CD206^+^CCL18^+^ macrophages and CD3^+^CD8^+^CD56-XCL1^+^ T cells were determined by X-tile program.

### Flow cytometry analysis

Fresh paired tumor and non-tumor tissues were obtained from another seven HCC patients who underwent hepatectomy in July 2019. None of these patients had received anti-tumor treatments before surgery. Fresh tissues were minced into pieces and digested in RPMI-1640 medium (Gibcol, 11875093) containing 1 mg/mL collagenase IV (Gibcol, 17104019) and 0.4 mg/mL hyaluronidase mixture on a gentleMACSTM Octo Dissociator with Heaters machine (Miltenyi Biotec, 130-096-427) for 1 h at 37 °C. Cell suspensions were filtered through a 400-mesh sieve and mononuclear leukocytes were obtained by Ficoll density gradient centrifugation. Flow cytometry was performed on a BD LSR Fortessa cell analyzer (BD Bioscience) according to the manufacturer’s instructions and analyzed by FlowJo software version 9.3.2.

### TCGA data analysis

The TCGA-LIHC data were used to evaluate the prognostic effect of individual genes or gene sets derived from specific cell clusters. Patient cohorts were grouped into high and low expression groups by the median value of the normalized average expression of strong marker genes (logFC > 2). Kaplan–Meier survival curves and *P* values were generated by R package survminer.

### Single-cell regulatory network inference and clustering analysis

Gene regulatory networks were identified using SCENIC^27^ (v1.1.0) with default settings. To reduce the computing time, a python implementation in SCENIC (GRNBoost) was used.

### Developmental trajectory inference

Monocle (v2.12.0)^23^ was applied to determine the potential lineage differentiation within each cell type. Only top 1000 variable genes identified by differentialGeneTest were selected for constructing the developmental tree.

### Cell–cell interaction

CellphoneDB^37^ (v1.1.0) was applied to identify cell-cell interactions for cells from the tumor and normal liver separately. For those significant interactions (*P* < 0.05), we also required the ligand/receptors were expressed in more than 5% corresponding cells. We compared interaction pairs between tumor and peri-tumor and filtered the interaction pairs specific for tumor.

### Statistical analysis

Statistical analysis was performed with the R, SPSS (v22, IBM, Armonk, NY) and Prism 6.0 (SanDiego, CA) softwares. Comparisons were performed using *χ*^2^ test, paired *t*-test or two-sided Wilcoxon rank-sum test. The cumulative survival time was estimated by Kaplan–Meier estimator with log-rank test. *P* values < 0.05 were considered statistically significant.

## Supplementary information

Supplementary Figures and Tables

Supplementary Table S3

Supplementary Table S6

Supplementary Table S7

## Data Availability

The raw sequencing data have been deposited in the Genome Sequence Archive (Genomics, Proteomics & Bioinformatics 2017) in BIG Data Center (Nucleic Acids Res 2019), Beijing Institute of Genomics (BIG), Chinese Academy of Sciences, under accession number: CRA002308 that are publicly accessible at https://bigd.big.ac.cn/gsa. All the other data generated in this study are included in the article and the additional files.

## Supplementary information

**Supplementary Information** accompanies the paper at (10.1038/s41421-020-00214-5).
