# Supplementary material for: Global immune characterization of HBV/HCV-related hepatocellular carcinoma identifies macrophage and T-cell subsets associated with disease progression
Source: Cell Discov. 2020 Dec 8;6:90. doi: 10.1038/s41421-020-00214-5 (PMC7721904; doi:10.1038/s41421-020-00214-5)
Supplement: Supplementary file 1 — Supplementary Figures and Tables [file 41421_2020_214_MOESM1_ESM.docx]

**Supplementary Figures and Tables**


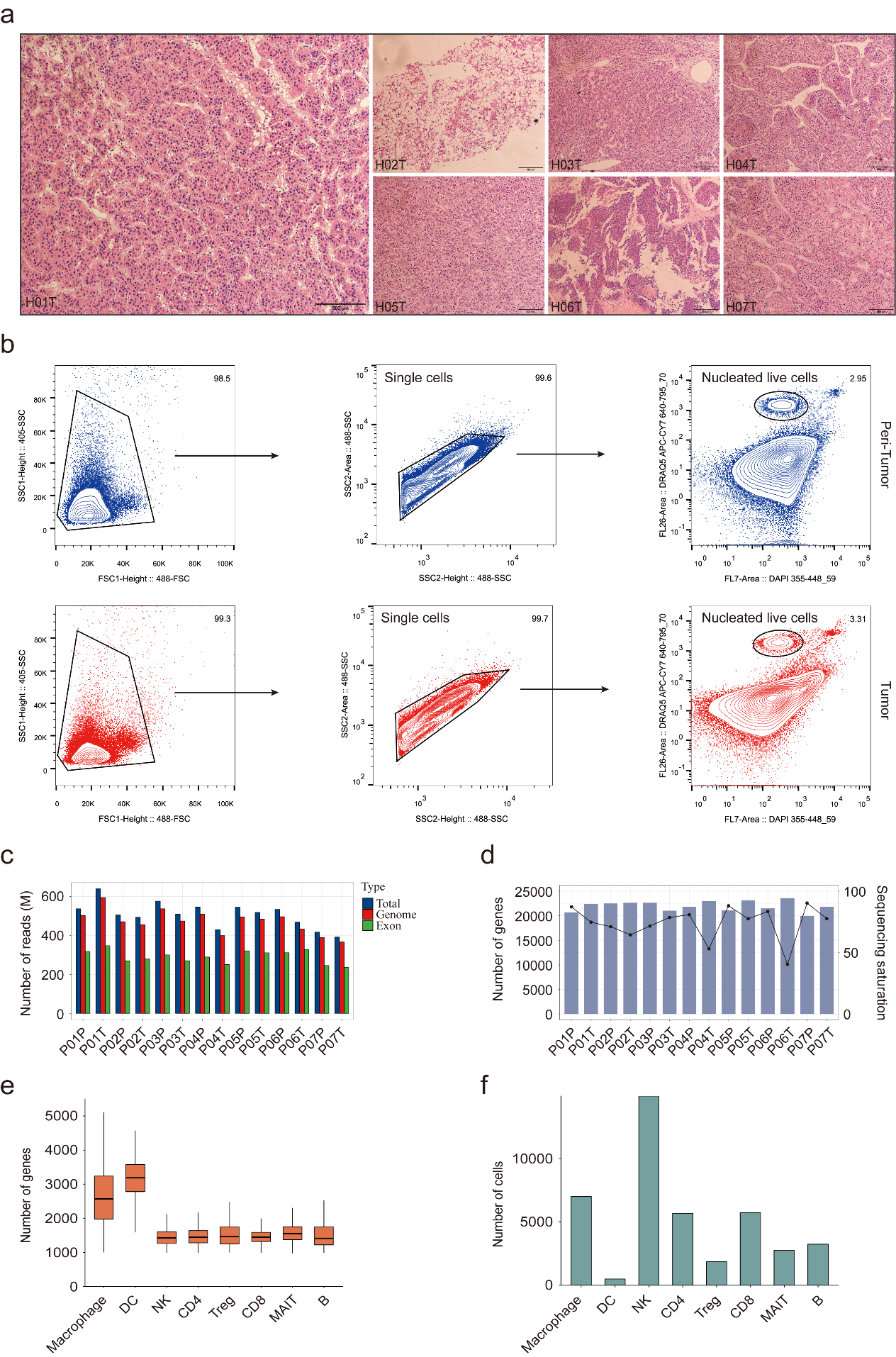


**Supplementary** Fig. S1 Single cell sorting and basic information of the single cell RNA-seq data. **a** Hematoxylin and eosin staining results of the tumor and peri-tumor tissues from each patient. Scale bar, 500μm. **b** Representative flow cytometry plots showing sorting strategy after doublet exclusion. All nucleated live cells were sorted from peri-tumor (up) and tumor tissues (down) by DRAQ5+DAPI-. **c** Number of detected reads from each patient, P and T represent the peri-tumor and tumor. **d** Number of detected genes and sequencing saturation curve of 7 HCC. **e** Box plots of number of genes detected across eight subsets of immune cells. Each box represents 0.25–0.75 percentile of gene count with line extension to 0.1-0.9 percentile; dot represents the mean gene count. **f** Number of cells in each type of immune cell.


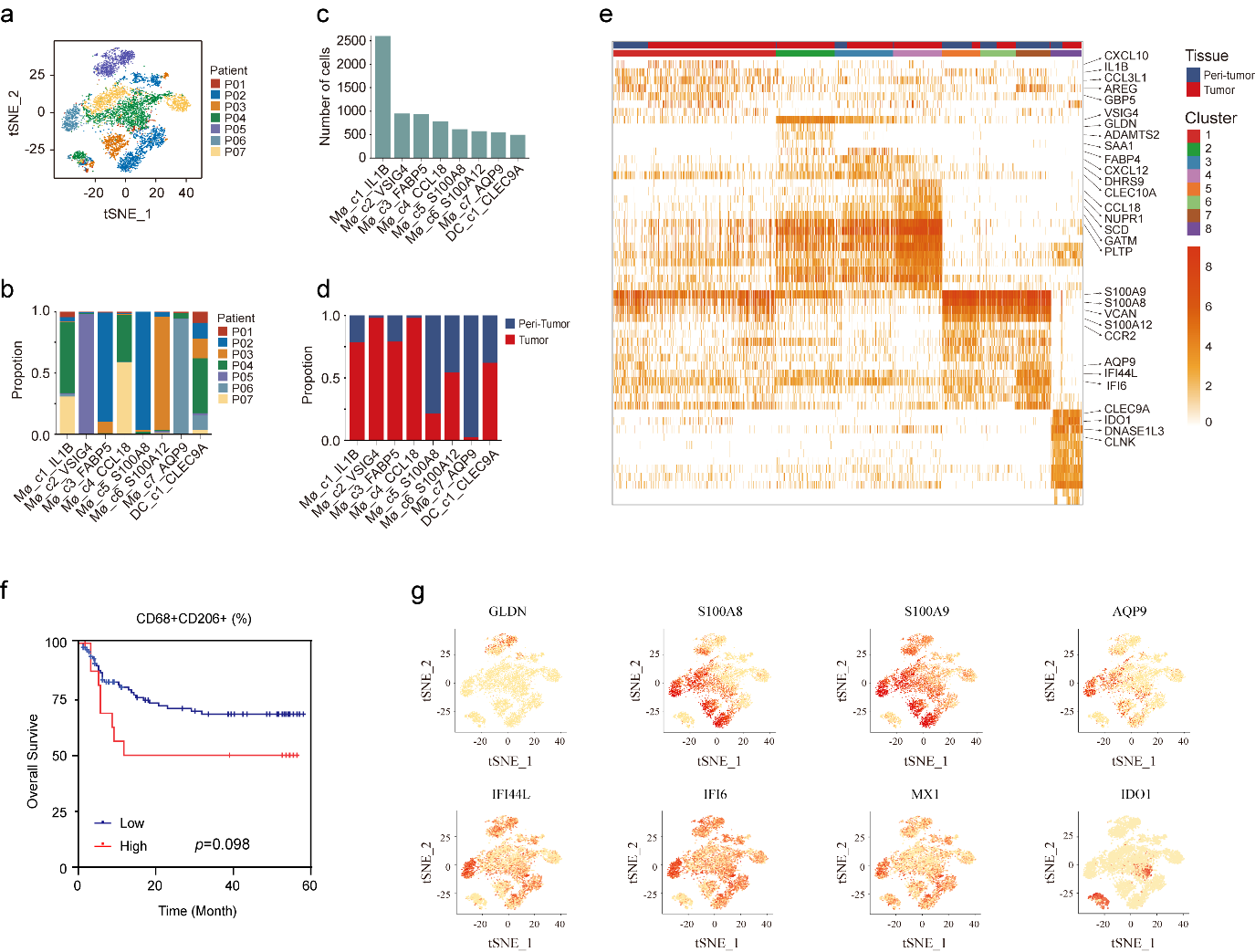


**Supplementary** Fig. S2 Origin and statistical information of eight clusters of **myeloid cells**. **a** t-SNE plots of macrophages from seven HCC patients annotated by the corresponding patient. **b** The proportion of eight clusters in each patient. **c** Number of cells in each subsets of myeloid cells. **d** The percentages of myeloid cells in each cluster of peri-tumor and tumor tissues. **e** Gene expression heat map of 8 macrophage clusters. Rows represent signature genes and columns represent different clusters. **f** Kaplan–Meier analyses of the correlation between proportion of CD68+CD206+ macrophage and the overall survival in patients with HCC (*p* = 0.098, Log-rank test). **g** t-SNE plot of expression levels of selected genes in different clusters of myeloid cells.


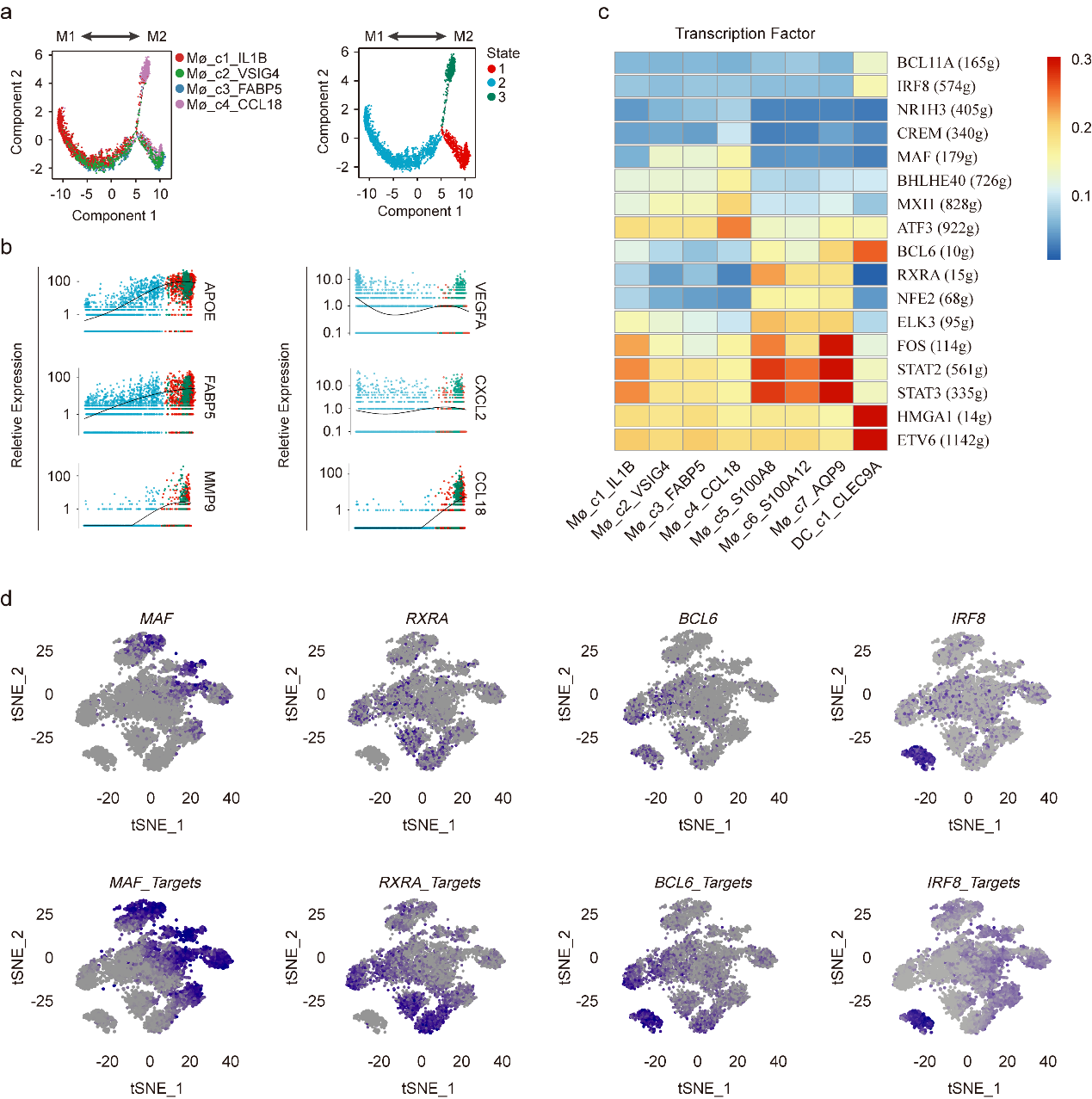


**Supplementary** Fig. S3 Transition and expression regulation of different **myeloid cell subsets**. **a** Pseudotime trajectory of macrophages with cluster Mø_c1, Mø_c2, Mø_c3, and Mø_c4 demonstrated in the trajectory (left), and the pseudotime trajectory was separated into three cell states (right). **b** Expression of dynamic genes along the pseudotime trajectory. **c** Heatmap of the expression regulation by transcription factors for each of the myeloid cells from 8 clusters. **d** t-SNE plots of the expression of selected genes, and for the AUC of the estimated regulon activity of these transcription factors, corresponding to the degree of expression regulation of their target genes.


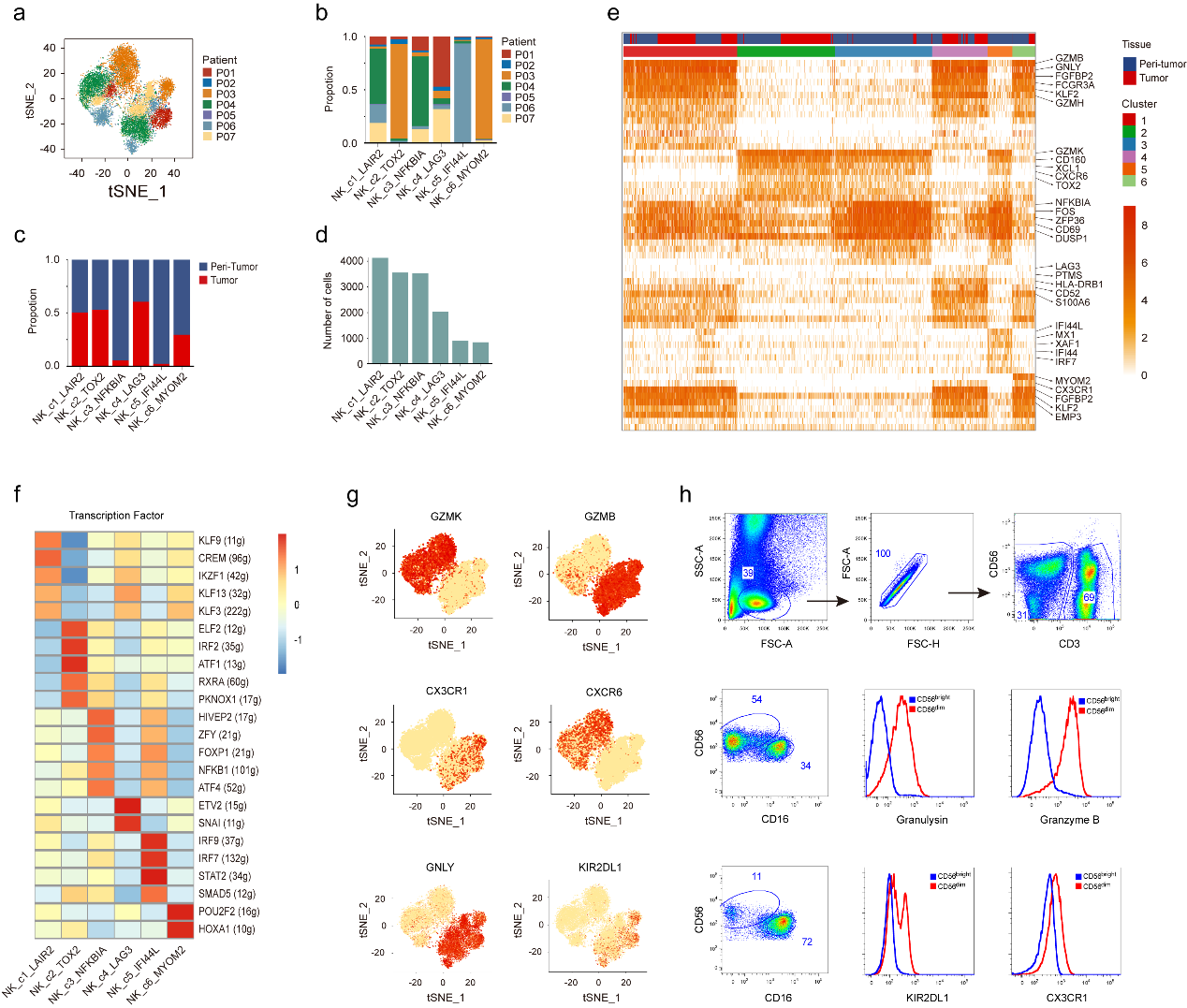


**Supplementary** Fig. S4 Transcriptome and expression regulation heterogeneity of NK cells. **a** t-SNE plots of NK cells from seven HCC patients annotated by the corresponding patient. **b** The proportion of six clusters in each patient. **c** The percentages of NK cells in each cluster of peri-tumor and tumor tissues. **d** Number of cells in each subsets of NK cells. **e** Gene expression heat map of six NK clusters. Rows represent signature genes and columns represent different clusters. **f** Heatmap of the expression regulation by transcription factors for each of the NK cells. **g** t-SNE plots of the expression of the indicated selected genes of NK clusters. **h** The protein levels of Granulysin, Granzyme B, KIR2DL1, and CX3CR1 in CD56^bright^ and CD56^dim^ NK subsets were assessed by flow cytometry.


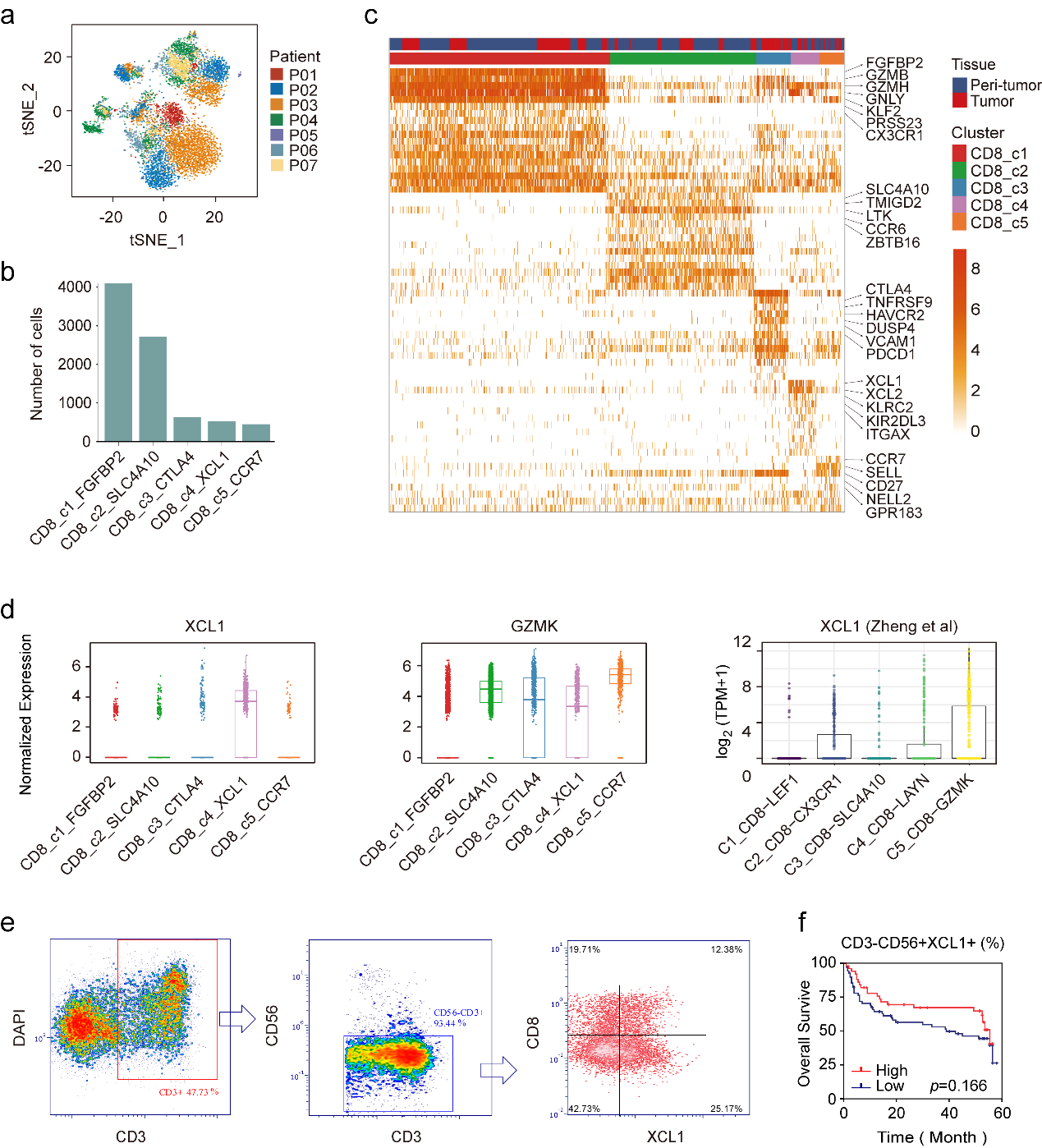


**Supplementary** Fig. S5 Five subsets of CD8+ T cells were identified in HCC TME. **a** Five distinct CD8+ T cell clusters from seven HCC patients were displayed with a t-SNE plot. **b** Number of cells in each subset of CD8+ T cells. **c** Gene expression heat map of five CD8+ T cell clusters. Rows represent signature genes and columns represent different clusters. **d** Boxplots of the expression of XCL1, GZMK in five subsets of CD8+ T cells or previous data (Zheng *et al* ^13)^. The horizontal line represents the median. **e** Strategies for defining cutoff values of CD3+CD8+CD56-XCL1+ T cells. f Kaplan–Meier analyses of the correlation between proportion of CD3-CD56+XCL1+ NK cells and the overall survival in patients with HCC **(***p* = 0.166, Log-rank test).


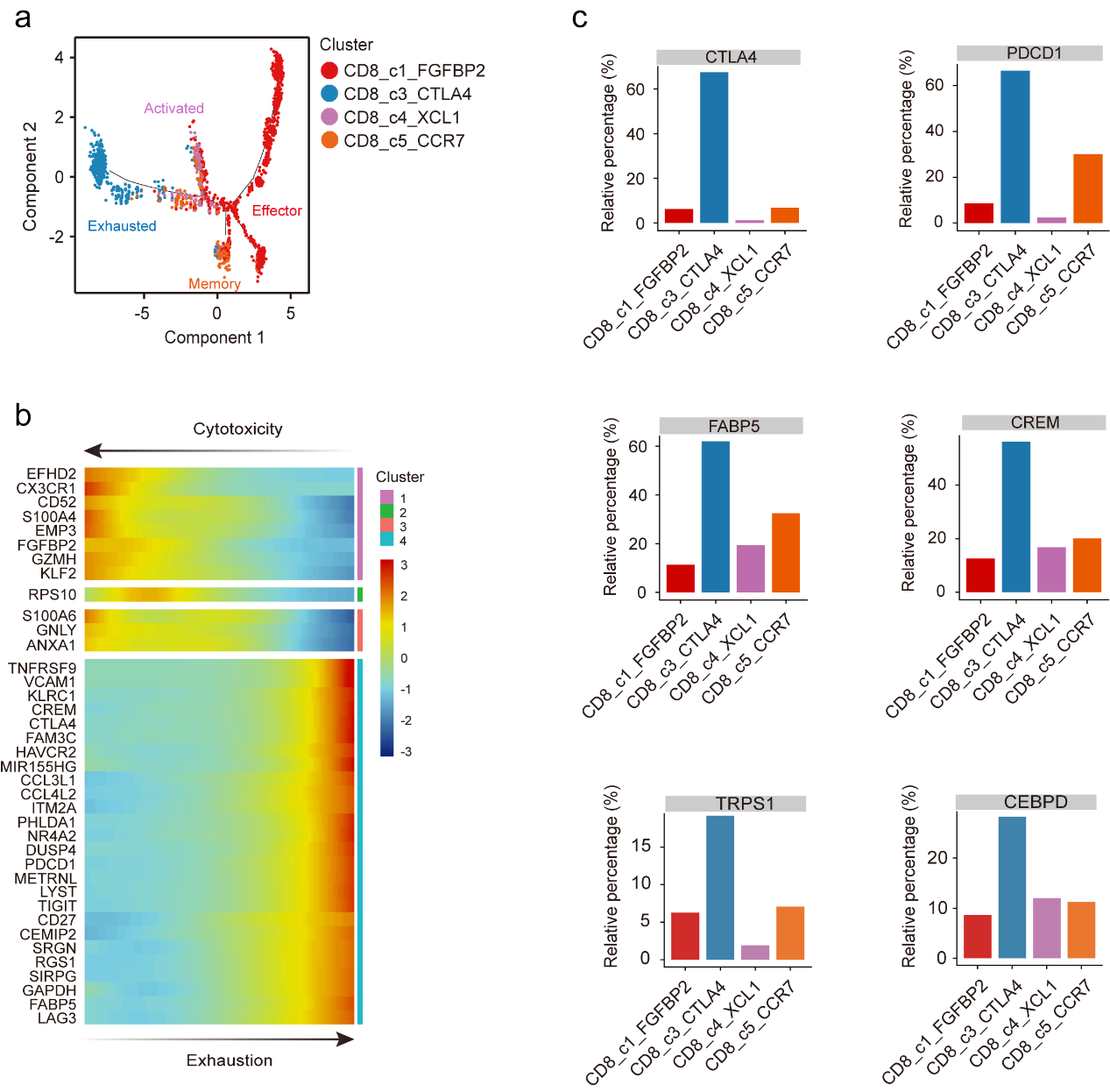


**Supplementary** Fig. S6 Transcriptome dynamics during pseudotime trajectory of four clusters of CD8+ T cells. **a** Pseudotime plot of CD8+ T cells (MAIT cells of CD8_c2 were removed) in a two-dimensional state space defined by Monocle 2. Each dot corresponds to a single cell and each color represents a cluster. **b** Heatmap showing scaled expression of dynamic genes along the pseudotime trajectory. **c** Representative histograms of selected gene expression along the trajectory.


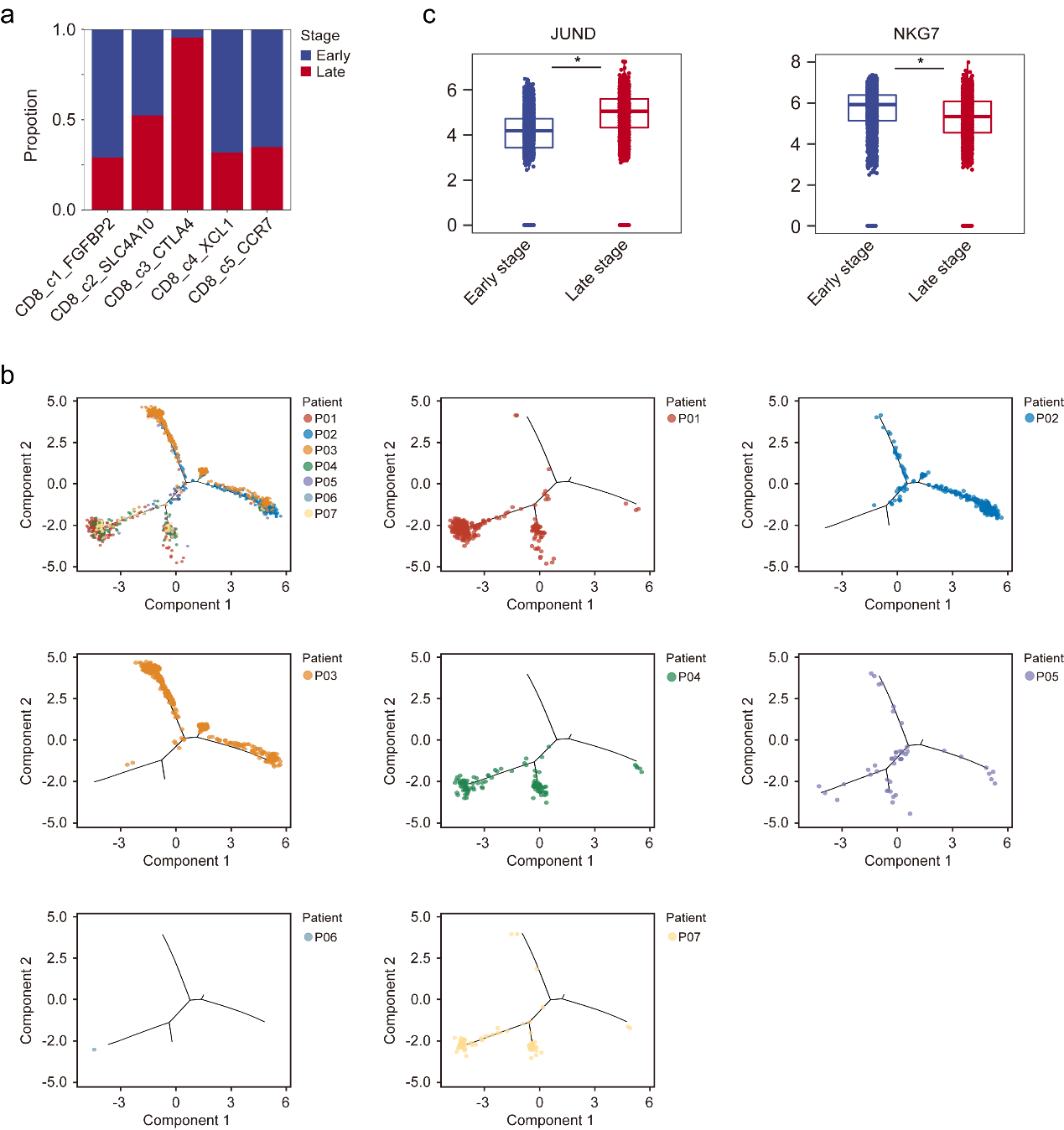


**Supplementary** Fig. S7 Transcriptome dynamics during pseudotime trajectory of CD8+ T cells in early stage or late stage HCC. **a** The percentages of cells in each cluster of early stage (P02, P03, and P05) or late stage (P01, P04, P06, and P07) HCC patients. **p* < 0.01. **b** Pseudotime trajectory of effector CD8+ T cells from each patient with all individual cluster demonstrated in the trajectory. **c** Expression of selected genes in early-stage and late-stage HCC are shown in the boxplots ( [wilcoxon rank sum test](http://www.baidu.com/link?url=_Ro4ZZoNssazoQ4BPU_rcUyZywKLlvPR6N_uqao9GnjIrakSgnc8IPMSVoWfTwbQBozP8lVR74e7rxXDMWdJR2PeqNWkP8YSqWKyjCAjM9Sooxbcx3ofzlyqfVIPHdAK) was used, all *p* values *<* 2.2×10^-16^).


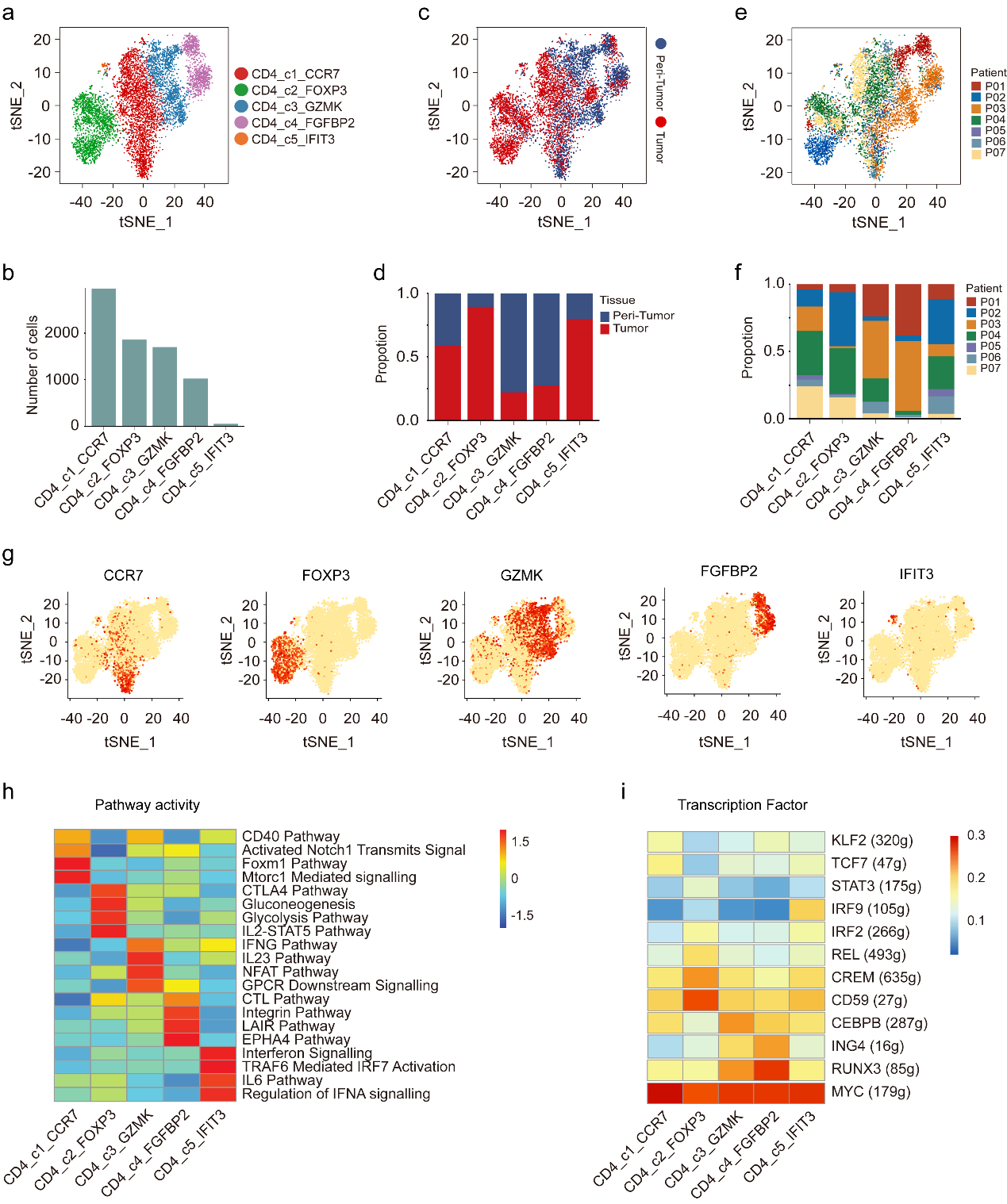


**Supplementary** Fig. S8 Transcriptome heterogeneity of CD4+ T cells. **a** Five subsets of CD4+ T cells were identified and shown in the t-SNE plot. **b** Number of cells in each subset of CD4+ T cells. **c** t-SNE plots of CD4+ T cells derived from peri-tumor and tumor tissues. **d** The proportion of CD4+ T cells derived from either peri-tumor or tumor site in each cluster. **e** t-SNE plots of CD4+ T cells from seven HCC patients annotated by the corresponding patient. **f** The proportion of five CD4+ T cell clusters in each patient. **g** t-SNE plots of the expression of the indicated marker genes of CD4+ T clusters. **h** Differences in pathway activities scored per cell by GSVA between the different CD4+T cell clusters. **i** Heatmap of the t values of AUC scores of expression regulation by transcription factors, as estimated using SCENIC, per CD4+ T cell cluster.


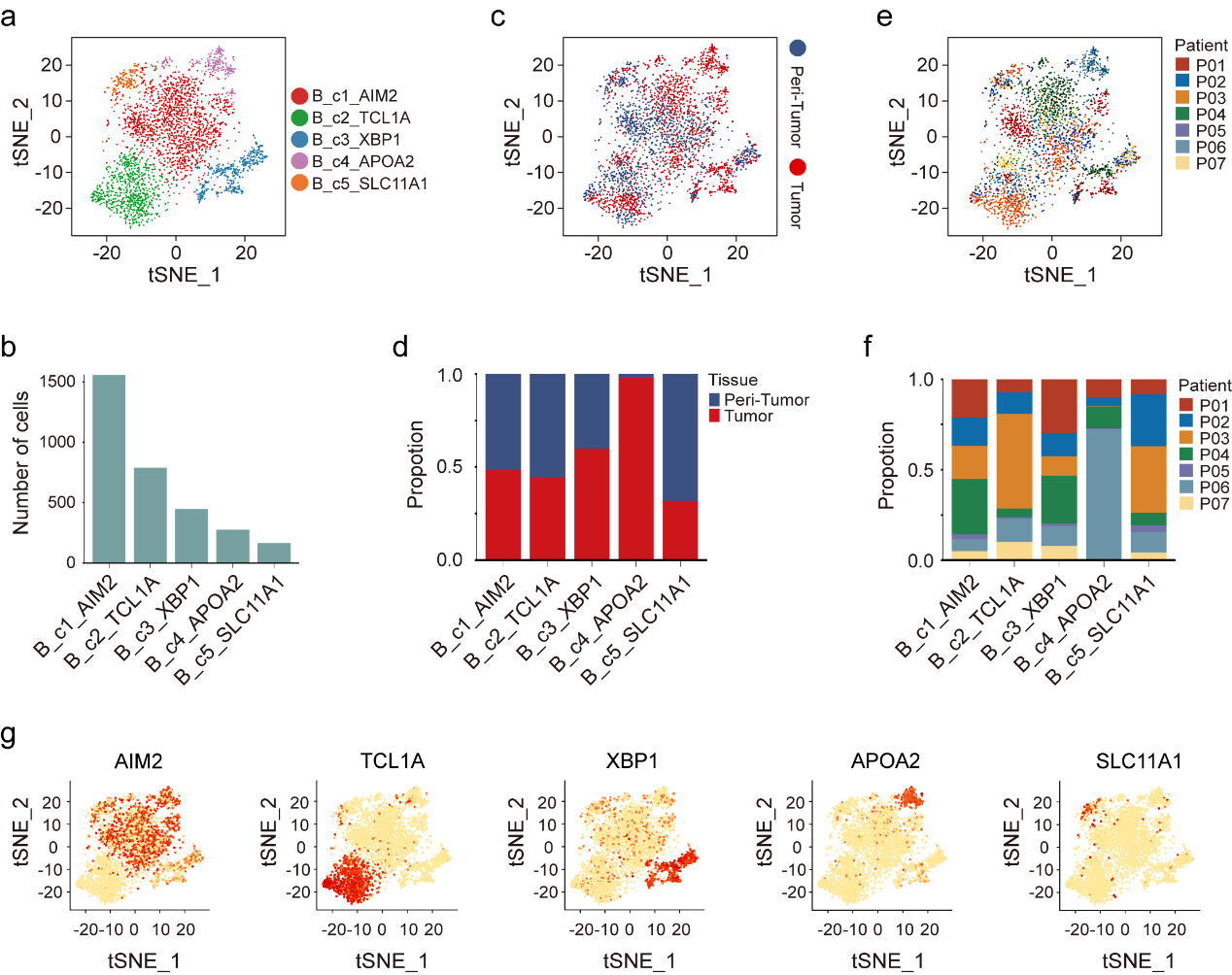


**Supplementary** Fig. S9 Five subsets of B cells were identified in HCC TME. **a** Five subsets of B cells were identified and shown in the t-SNE plot. **b** Number of cells in each subset of B cells. **c** t-SNE plots of B cells derived from peri-tumor and tumor tissues. **d** The proportion of B cells derived from either peri-tumor or tumor site in each cluster. **e** t-SNE plots of B cells from seven HCC patients annotated by the corresponding patient. **f** The proportion of five B cell clusters in each patient. **g** t-SNE plots of the expression of the indicated marker genes of B cell clusters.

| **Table S1.** **Clinicopathological characteristics of 7 HCC patients** | | | | | | | | | | | |
| --- | --- | --- | --- | --- | --- | --- | --- | --- | --- | --- | --- |
| **Patient_ID** | **Gender** | **Age** | **HBsAg** | **HBeAg** | **HBVDNA** | **HCVAb** | **AFP**  **(ng/ml)** | **Tumor size (cm)** | **Tumor differentiation** | **Liver cirrhosis** | **TNM stage (AJCC, 8th)** |
| P1 | Male | 69 | Positive | Negative | 194000 | Negative | 5.1 | 9, 1.5 | III | No | IIIA |
| P2 | Male | 67 | Positive | Negative | 7470 | Negative | 11.5 | 3 | II | Yes | I |
| P3 | Male | 45 | Positive | Negative | Negative | Negative | 2.8 | 7 | II | No | I |
| P4 | Male | 60 | Positive | Positive | 749 | Negative | 5021 | 7, 1.5 | III | Yes | IIIA |
| P5 | Male | 59 | Negative | Negative | Negative | Positive | 3.1 | 7 | II-III | No | I |
| P6 | Male | 36 | Positive | Negative | 127000 | Negative | >60500 | 11 | III | Yes | IIIB |
| P7 | Male | 58 | Positive | Negative | 1650 | Negative | >60500 | 10.5 | III | No | IIIB |

| **Table S2. Basic information of cells detected by sc-RNA seq in 7 HCC patients** | | | | | | | | | | | | |  |
| --- | --- | --- | --- | --- | --- | --- | --- | --- | --- | --- | --- | --- | --- |
| **Celltype** | **Genes** | **UMIs** | **Cells** | **P01** | **P02** | **P03** | **P04** | **P05** | **P06** | **P07** | **Peri-tumor** | **Tumor** | |
| **MAC** | 2573 | 8669 | 7008 | 147 | 1551 | 656 | 1840 | 961 | 572 | 1281 | 2073 | 4935 | |
| **DC** | 3187 | 16438 | 496 | 45 | 64 | 80 | 222 | 9 | 58 | 18 | 188 | 308 | |
| **NK** | 1424 | 3392 | 14934 | 1832 | 398 | 4278 | 4654 | 57 | 1762 | 1953 | 9303 | 5631 | |
| **CD4** | 1444 | 4453 | 5677 | 918 | 482 | 1784 | 1288 | 111 | 310 | 784 | 3274 | 2403 | |
| **Treg** | 1460 | 3814 | 1856 | 113 | 742 | 27 | 629 | 17 | 35 | 293 | 197 | 1659 | |
| **CD8** | 1446 | 3945 | 5727 | 656 | 1344 | 2174 | 800 | 72 | 418 | 263 | 3306 | 2421 | |
| **MAIT** | 1552 | 4489 | 2760 | 91 | 584 | 649 | 616 | 82 | 219 | 519 | 2110 | 650 | |
| **B** | 1411 | 4704 | 3240 | 565 | 451 | 811 | 674 | 66 | 470 | 203 | 1540 | 1700 | |
| Number of genes/UMIs/cells in different immune subsets, patients, and tissues. | | | | | | | | | | | |  | |

**Table S4.** **Correlation between proportion of CD68+CD206+CCL18+ macrophage and CD3+CD8+CD56-XCL1+ T cells and clinicopathological characteristics of HCC patients**

| **Characteristics** | **Total**  **(n = 116)** | **Proportion of CD3+CD8+CD56-XCL1+** | | **P-value*** | **Total**  **(n = 121)** | **Proportion of CD68+CD206+CCL18+** | | **P-value*** |
| --- | --- | --- | --- | --- | --- | --- | --- | --- |
|  |  | **Low**  **(n = 85)** | **High**  **(n = 31)** |  |  | **Low**  **(n = 88)** | **High**  **(n = 33)** |  |
| **Age** |  |  |  |  |  |  |  |  |
| ≤51 | 54 | 37 | 17 | 0.280 | 58 | 44 | 14 | 0.458 |
| >51 | 62 | 48 | 14 |  | 63 | 44 | 19 |  |
| **Gender** |  |  |  |  |  |  |  |  |
| Male | 100 | 74 | 26 | 0.892^†^ | 106 | 76 | 30 | 0.714^†^ |
| Female | 16 | 11 | 5 |  | 15 | 12 | 3 |  |
| **HBsAg** |  |  |  |  |  |  |  |  |
| Negative | 3 | 3 | 0 | 0.690^†^ | 3 | 1 | 2 | 0.371^†^ |
| Positive | 113 | 82 | 31 |  | 118 | 87 | 31 |  |
| **AFP (ng/ml)** |  |  |  |  |  |  |  |  |
| ≤20 | 45 | 35 | 10 | 0.383 | 47 | 36 | 11 | 0.446 |
| >20 | 71 | 50 | 21 |  | 74 | 52 | 22 |  |
| **ALT (U/L)** |  |  |  |  |  |  |  |  |
| ≤75 | 105 | 78 | 27 | 0.688^†^ | 110 | 79 | 31 | 0.723^†^ |
| >75 | 11 | 7 | 4 |  | 11 | 9 | 2 |  |
| **Liver cirrhosis** |  |  |  |  |  |  |  |  |
| No | 8 | 7 | 1 | 0.597^†^ | 9 | 6 | 3 | 0.972^†^ |
| Yes | 108 | 78 | 30 |  | 112 | 82 | 30 |  |
| **Tumor size (cm)** |  |  |  |  |  |  |  |  |
| ≤5 | 64 | 43 | 21 | 0.152 | 79 | 63 | 16 | **0.025** |
| >5 | 52 | 42 | 10 |  | 42 | 25 | 17 |  |
| **Tumor number** |  |  |  |  |  |  |  |  |
| Single | 101 | 72 | 29 | 0.346^†^ | 106 | 79 | 27 | 0.237 |
| Muitiple | 15 | 13 | 2 |  | 15 | 9 | 6 |  |
| **Lymph node metastasis** |  |  |  |  |  |  |  |  |
| No | 115 | 84 | 31 | 1.000^†^ | 120 | 87 | 33 | 1.000 |
| Yes | 1 | 1 | 0 |  | 1 | 1 | 0 |  |
| **Tumor differentiation** |  |  |  |  |  |  |  |  |
| I-II | 83 | 27 | 56 | **0.045**^†^ | 84 | 65 | 19 | 0.083 |
| III-IV | 33 | 4 | 29 |  | 37 | 23 | 14 |  |
| **TNM stage** |  |  |  |  |  |  |  |  |
| I+II | 71 | 49 | 22 | 0.193 | 99 | 76 | 23 | **0.034** |
| III+IV | 45 | 36 | 9 |  | 22 | 12 | 10 |  |

Abbreviations: HCC, hepatocellular carinoma; HBsAg, hepatitis B surface antigen; AFP, α-fetoprotein; ALT, alanine transaminase; TNM, tumor-nodes-metastases. The same cohort of tissue microarray (TMA) was used for these two experiments. Five cores were lost in CD3+CD8+CD56-XCL1+ cohort. Number in bold indicate that the *p* value is significant. *Pearson Chi-square test. ^†^Chi-square with Yates’ correction.

| **Table S5. Signature genes of M1 and M2 macrophage** | |
| --- | --- |
| **M1** | **M2** |
| iNOS | ARG1/2 (Arginase) |
| IL12 | IL10 |
| CD64 (FcγR1A) | CD32 |
| CD64 (FcγR1B) | CD163 |
| CD64 (FcγR1C) | CD23 (FCER2) |
| CD80 (B7-1) | CD200R1 |
| CXCR10 | PD-L2 (PDCD1LG2) |
| IL23 | PD-L1 (CD274) |
| CXCL9 | MARCO |
| CXCL10 | CSF1R |
| CXCL11 | CD206 (MRC1) |
| CD86 (B7-2) | Il1RA (IL1RN) |
| IL1A | Il1R2 |
| IL1B | IL4R |
| IL6 | CCL4 |
| TNFa | CCL13 |
| MHCII | CCL20 |
| CCL5 | CCL17 |
| IRF5 | CCL18 |
| IRF1 | CCL22 |
| CD40 | CCL24 |
| IDO1 | LYVE1 |
| KYNU | VEGFA |
| CCR7 | VEGFB |
|  | VEGFC |
|  | VEGFD |
|  | EGF |
|  | Cathepsin A (CTSA) |
|  | CTSB |
|  | CSTC |
|  | CTSD |
|  | TGFB1 |
|  | TGFB2 |
|  | TGFB3 |
|  | MMP14 |
|  | MMP19 |
|  | MMP9 |
|  | CLEC7A |
|  | WNT7b |
|  | FASL |
|  | TNFSF12 |
|  | TNFSF8 |
|  | CD276 (B7-H3) |
|  | VTCN1 (BH-H4) |
|  | MSR1 (CD204) |
|  | FN1 |
|  | IRF4 |

**Table S8. Anti-human antibodies used in flow cytometry (FCM) and multiplex Immunohistochemistry (mIHC)**

| **Ant­ibody** | **Channel** | **Catalogue or clone** | **Company** |
| --- | --- | --- | --- |
| CD3 | BV786 | SK7 | BD |
| CD11b | BV785 | M1/70 | Biolegend |
| CD14 | PECy7 | HCD14 | Biolegend |
| CD16 | PECy7 | 3G8 | BD |
| CD45 | PB | HI30 | Biolegend |
| CD56 | BV711 | HCD56 | Biolegend |
| KIR2DL1 | FITC | 556062 | BD |
| CX3CR1 | APC | 2A9-1 | Biolegend |
| CD206 | A700 | 321132 | Biolegend |
| GranzymeB | PE/Dazzle594 | QA16A02 | eBioscience |
| Granulysin | PE | GM26E7 | Biolegend |
| XCL1 |  | Ab112494 | Abcam |
| CD3 |  | ab16669, SP7 | Abcam |
| CD8 |  | M7103, C8/144B | Dako |
| CD56 |  | 99746 | CST |
| CCL18 |  | Ab104867 | Abcam |
| CD68 |  | 76437 | CST |
| CD206 |  | 91992 | CST |
| CREM |  | A5624 | Abclonal |
| DRAQ5 |  |  | CST |
| DAPI |  |  | Biolegend |

Table S3. Marker genes for clusters in each cell type.

Table S6. Differential expression genes between early stage and advanced HCC.

Table S7. Cell-cell interactions that presented specifically in HCC tumors.
